# New Mechanistic Evidence for Perfluorodecanoic Acid (PFDA) Teratogenicity via CYP26A1-Mediated Retinoic Acid Metabolism and Signaling

**DOI:** 10.1021/acs.chemrestox.5c00468

**Published:** 2026-03-30

**Authors:** Michaela Hvizdak, Sylvie E. Kandel, Jed N. Lampe

**Affiliations:** Department of Pharmaceutical Sciences, Skaggs School of Pharmacy, 12226 University of Colorado, Aurora, Colorado 80045, United States

## Abstract

Craniofacial abnormalities account for roughly one-third of all congenital birth defects worldwide. A growing body of evidence suggests that *per*- and polyfluoroalkyl substances (PFAS) are teratogenic in humans and laboratory animals, causing craniofacial morphological defects. PFAS structurally resemble the natural ligands of cytochrome P450 (CYP) enzymes involved in neonatal development, including the morphogen all-*trans*-retinoic acid (*at*RA). *at*RA regulates over 500 target genes during embryogenesis, including those related to craniofacial development. During pregnancy, circulating *at*RA concentrations are tightly maintained at the low nanomolar level. The fetus cannot synthesize *at*RA *de novo*, nor can the fetal liver reliably clear excess morphogen entering from maternal circulation to meet physiological demands. Therefore, maternal *at*RA homeostasis is paramount to proper fetal growth and development. In adults, members of the CYP26 family play a primary role in *at*RA clearance, including CYP26A1. PFAS disruption of maternal hepatic *at*RA metabolism via CYP26 may represent one pathological mechanism for the significant birth defects associated with prenatal exposure. We performed an *in vitro* screening of 13 prominent PFAS to measure their potential to inhibit CYP26A1 and CYP26B1 metabolism of *at*RA. Of the PFAS tested, PFDA was the most potent inhibitor of CYP26A1, with half-maximal inhibitory concentrations of 49.5 and 51.3 μM for 4-hydroxy- and 4-oxo-retinoic acid metabolite formation, respectively. No significant inhibition of CYP26B1 was observed. PFDA additionally perturbed *at*RA metabolism and signaling in female primary human hepatocytes following 48 h semistatic incubations. Based on our data, the *at*RA metabolic pathway through CYP26A1 regulation is a target for prenatal PFDA exposure, likely invoking irreversible consequences for the vulnerable fetus and neonate.

## Graphical abstract

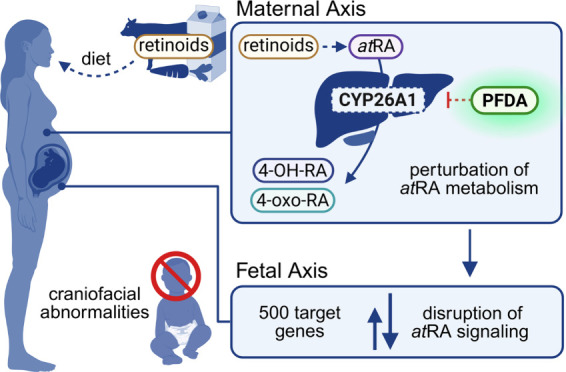

## Introduction

Craniofacial abnormalities comprise approximately one-third of all congenital birth defects and are a significant threat to infant mortality.^1^ Prenatal exposure to *per-* and polyfluoroalkyl substances (PFAS) is associated with significant morphological defects, including craniofacial abnormalities in both humans and laboratory animals.^2–5^ Other adverse pregnancy outcomes, including reduced birth weight and body length, have also been reported in neonates following gestational exposure to PFAS.^6–9^ Structurally, PFAS resemble some of the native ligands of cytochrome P450 (CYP) enzymes, including short-chain fatty acids. PFAS have been shown to inhibit the activities of drug-metabolizing CYPs, including CYP2D6, CYP3A4, and CYP3A7.^10–12^ Our lab previously demonstrated the capacity for PFAS to bind to human neonatal CYP3A7 and inhibit its oxidative activity, including hydroxylation of the endogenous substrate and steroid dehydroepiandrosterone sulfate (DHEA-S).^12^ Inhibition of DHEA-S metabolism disrupts estriol biosynthesis during pregnancy, potentially contributing to the preterm birth and reduced body weight phenotypes observed with PFAS exposure. However, there continue to be marked gaps in the scientific literature elucidating the mechanisms between developmental PFAS exposure and craniofacial abnormalities in neonates.

A major pathway involved in regulating the formation of the head and face surrounds retinoic acid metabolism and signaling.^13–15^ Throughout gestation, the fetus relies entirely on maternal delivery of vitamin A derivatives, including retinoic acid obtained from the diet or hydrolyzed from retinyl esters stored in the liver, as natural retinoids cannot be synthesized *de novo*.^16,17^ All-*trans*-retinoic acid (*at*RA; tretinoin) is the most predominant retinoic acid, and the most biologically active isomer compared to 9-*cis*-retinoic acid (9-*cis*-RA; alitretinoin) and 13-*cis*-retinoic acid (13-*cis*-RA; isotretinoin).^18,19^ *at*RA is an important signaling molecule and morphogen that regulates over 500 target genes involved in germ layer and body axis formation, hindbrain patterning, cardiogenesis, and eye development.^18,20–22^ During embryogenesis, *at*RA interacts with the nuclear hormone retinoic acid receptor (RAR; α, β, and γ), which exists as a heterodimer with retinoid X receptor (RXR; α, β, and γ; activated by 9-*cis*-RA and polyunsaturated fatty acids), bound to retinoic acid response elements (RAREs) on promoters of target genes, inducing a conformational change that allows for transcriptional activation.^20,21,23^ Circulating plasma *at*RA concentrations are tightly controlled at ∼2–11 nM during pregnancy in humans through an autoregulatory feedback mechanism, in which excess *at*RA induces transcription of genes coding for retinoic acid hydroxylases, including CYP26A1.^24–26^

CYP26A1 is the most prominent of the three members belonging to the CYP26 retinoic acid hydroxylase family (CYP26A1, -B1, and -C1), and is expressed in the liver and placenta.^27,28^ Retinoic acid hydroxylases catalyze the oxidation of *at*RA to form the primary biologically active metabolites 4-hydroxy-retinoic acid ((4*R*) and (4*S*)-OH-RA) and, subsequently, 4-oxo-retinoic acid (4-oxo-RA), among other minor metabolites (Figure 1).^28,29^ Although formation of 4-oxo-RA may arise from both 4-OH-RA enantiomers, it is the major product of (4*R*) and the minor product of (4*S*)-OH-RA, relative to other dihydroxylated products.^30^ Other metabolites include 16-OH and 18-OH-RA, of which formation is highly dependent on the specific enzyme isoform.^31^ In addition to the CYP26 enzyme family, secondary retinoic acid hydroxylases include members of the CYP3A family, including CYP3A4/5 and CYP3A7 in the adult and fetal/neonatal livers, respectively, along with the CYP2C family.^28,29^ However, fetal liver retinoic acid hydroxylase expression and activity levels are insufficient in producing a protective maternal-fetal barrier against excess *at*RA.^32^ This makes the fetus particularly vulnerable to maternal disruptions in CYP26A1-mediated *at*RA metabolism, which can cause severe morphological consequences in the neonate, including craniofacial abnormalities.^13,14^

**Figure 1:**
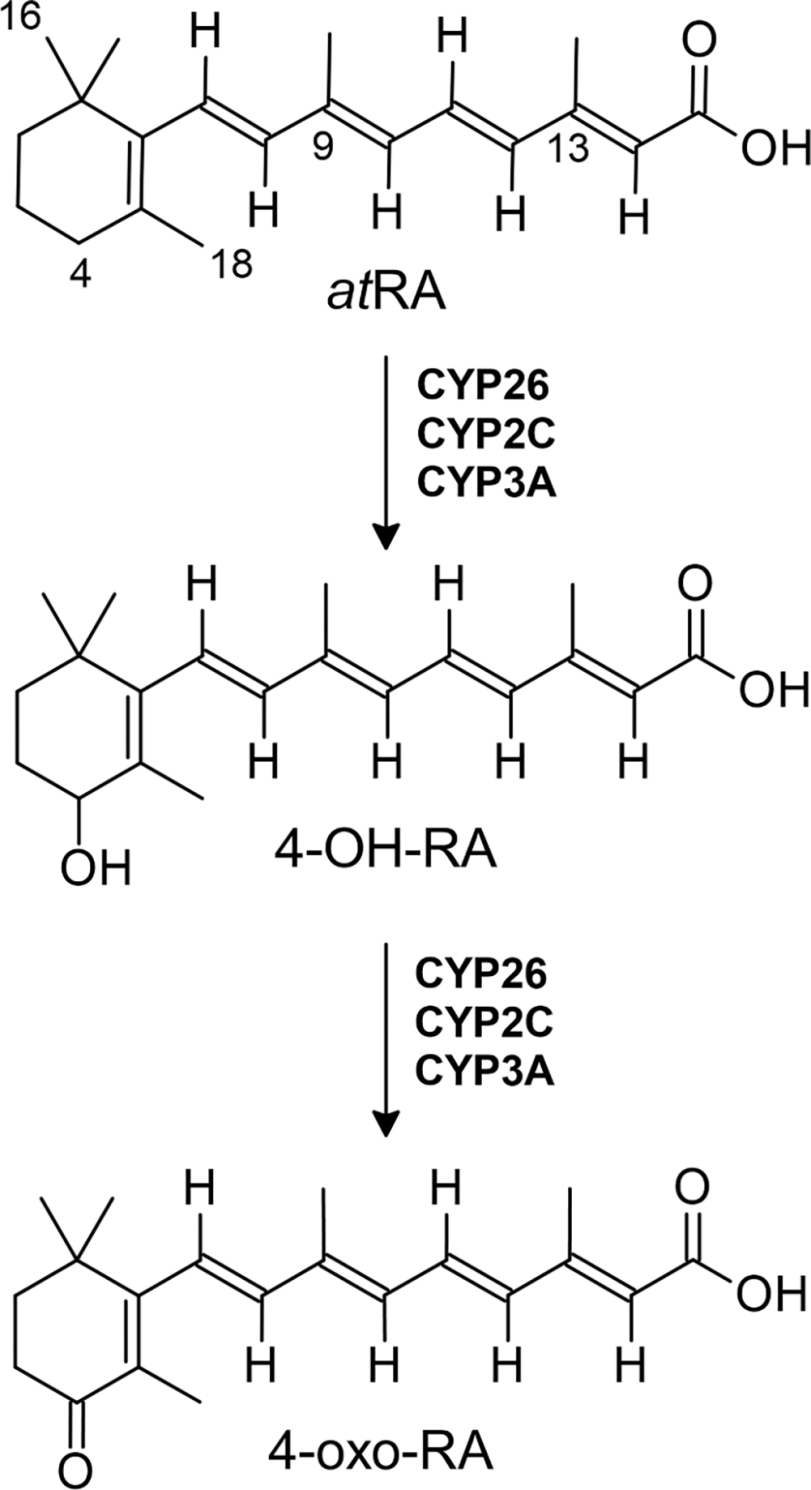
All-*trans*-retinoic acid (*at*RA) metabolism by human retinoic acid hydroxylases. Members of the CYP26, CYP2C, and CYP3A families perform two successive oxidations at the fourth position of the β-ionone ring, forming primary biologically active metabolites: 4-OH and 4-oxo-RA. Select carbons are numbered on *at*RA. Abbr.: *at*RA (all-*trans*-*retinoic* acid); 4-OH-RA (4-hydroxy-retinoic acid); 4-oxo-RA (4-oxo-retinoic acid).

PFAS are an anthropogenic class of nearly 15,000 ubiquitous aliphatics known for their carbon–fluorine bonds (C_*n*_F_2*n*+1_R), which are among the strongest bonds in organic chemistry.^33,34^ Classified as persistent organic pollutants (POPs), PFAS are not known to degrade in the environment through typical pathways. Although some may undergo physicochemical conversions that shorten their alkyl chains, most PFAS are themselves metabolically inert under normal physiological conditions.^35,36^ In the United States, drinking water contaminated with PFAS is a significant route of human exposure.^37,38^ In adults, major target organs include the liver, lungs, kidneys, and placenta.^39–41^ PFAS are known endocrine-disrupting chemicals (EDCs) due to their ability to interfere with endogenous hormone signaling and fatty acid homeostasis. Developing fetuses are also exposed to PFAS *in utero* via umbilical cord blood, where the compounds traverse the placental barrier and bioaccumulate in fetal tissues, including the lung, liver, heart, and central nervous system.^42,43^

In the latter part of the 20th century, private surveillance of occupational exposures to PFAS at DuPont revealed that two children born to employees in 1979–81 had major birth defects featuring craniofacial abnormalities, including malformed or missing eyes and nostrils.^44^ These were among the first reports identifying PFAS as potential teratogens, and they contributed to a federal phase-out process of historically used long-chain PFAS compounds, including perfluorooctanoic acid (PFOA) and its sulfonated counterpart, perfluorooctanesulfonic acid (PFOS), across the United States.^44^ These long-chain "legacy" PFAS have since been replaced with short-chain "emerging" PFAS, which were originally thought to be biologically inert.^11,45^ Despite global bans and increased regulations on the commercial uses of specific legacy compounds, 98% of the United States population has detectable serum levels of PFAS.^46,47^

Many of the birth defects and phenotypes associated with prenatal PFAS exposure are consistent with the outcomes reported following a disruption of retinoic acid signaling and homeostasis during critical stages of fetal development.^13–15^ Our previous research demonstrated the capacity for PFAS to inhibit the homeostatic activity of human neonatal CYP3A7 *in vitro.* ^12^ Given that CYP3A7 and CYP26A1 are both retinoic acid hydroxylases, they likely share topographical similarities in their ligand-binding pockets, allowing the same compounds to bind to and interact with their respective heme irons in a comparable manner. We therefore hypothesized that PFAS disrupt maternal *at*RA metabolism and signaling via CYP26A1 inhibition, providing a potential mechanism explaining some of the birth defects, including craniofacial abnormalities, observed in affected neonates.

To test this hypothesis, we screened six long-chain (PFOA, PFOS, PFNA, PFDA, PFDS, and PFUnDA) and seven short-chain (FBSA, PFBS, PFPeA, GenX, FHxSA, PFHxS, and 4:2 FTS) PFAS for their capacity to inhibit the oxidative activities of CYP26A1, as well as CYP26B1, via liquid chromatography-tandem mass spectrometry (LC-MS/MS) using *at*RA as the substrate (Table 1). Half-maximal inhibitory concentrations (IC_50_s) were determined for compounds achieving over 50% inhibition in the initial screenings. Molecular docking was then employed to further investigate the specific interactions between the CYP26A1 active site and both retinoid and PFAS compounds. In addition, female primary human hepatocytes (femPHHs; ages 16–40) were utilized to faithfully recapitulate PFAS inhibition kinetics in an advanced model of retinoic acid homeostasis in the maternal liver. This research identifies a novel mechanism of PFAS teratogenicity in humans through the dysregulation of maternal retinoic acid hydroxylase activity and signaling, establishing the CYP26 family as a vulnerable PFAS target throughout neonatal development.

**Table 1:**
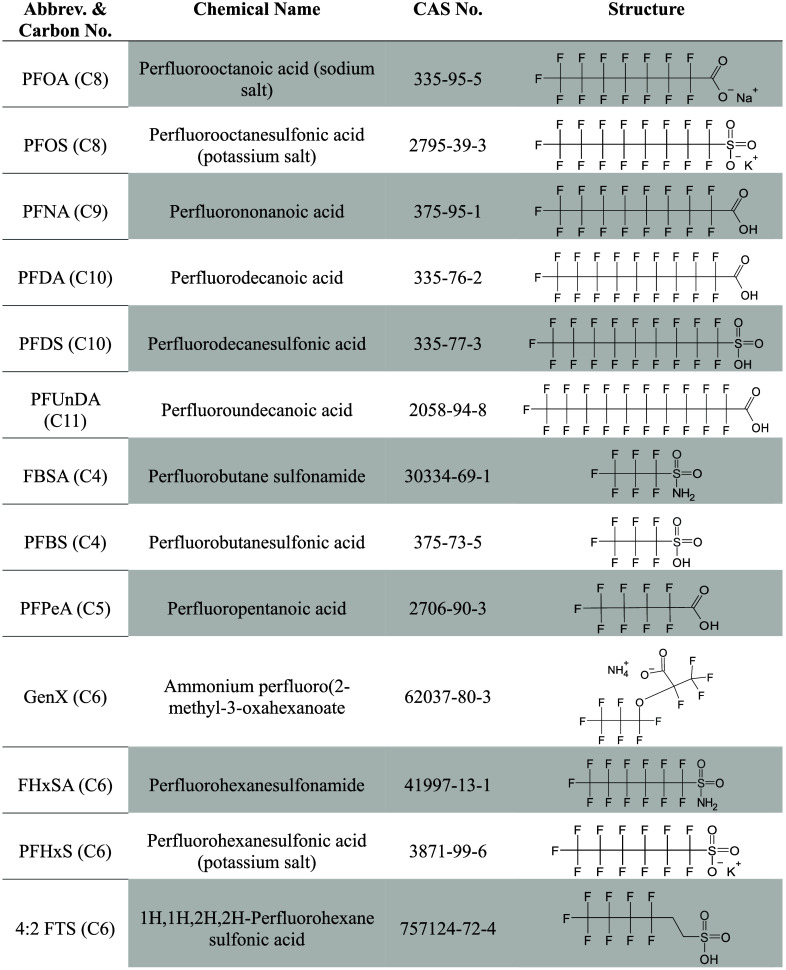
List of Relevant PFAS Candidates^a^

a Abbreviations and carbon number (abbrev. and carbon no.), chemical name, CAS number, and structure of PFAS compounds listed in the order of screening in the enzyme inhibition assay.

## Materials and Methods

### Chemicals and Test Systems

Perfluorooctanoic acid (PFOA; CAS: 335-95-5) was purchased from Thermo Fisher Scientific Inc. (Waltham, MA). Perfluorononanoic acid (PFNA; CAS: 375-95-1) and perfluorohexanesulfonic acid (PFHxS; CAS: 3871-99-6) were both acquired through Frontier Scientific Inc. (Logan, UT). Perfluorobutane sulfonamide (FBSA; CAS: 30334-69-1) and perfluorohexanesulfonamide (FHxSA; CAS: 41997-13-1) were purchased from AA Blocks (San Diego, CA). Ammonium perfluoro­(2-methyl-3-oxahexanoate) (GenX; CAS: 62037-80-3) was purchased from Manchester Organics (Runcorn, UK). 1*H*,1*H*,2*H*,2*H*-Perfluorohexanesulfonic acid (4:2 FTS; CAS: 757124-72-4) was acquired from Apollo Scientific (Bredbury, UK). Perfluorobutanesulfonic acid (PFBS; CAS: 375-73-5), perfluoropentanoic acid (PFPeA; CAS: 2706-90-3), perfluorooctanesulfonic acid (PFOS; CAS: 2795-39-3), perfluorodecanoic acid (PFDA; CAS: 335-76-2), perfluoroundecanoic acid (PFUnDA; CAS: 2058-94-8), and talarozole (TAL, R115866; CAS: 201410-53-9) were supplied by Sigma-Aldrich (St. Louis, MO). Perfluorodecanesulfonic acid (PFDS; CAS: 335-77-3), ketoconazole (KTC; CAS: 65277-42-1), the substrate all-*trans*-retinoic acid (*at*RA; CAS: 302-79-4), metabolite standards racemic 4-hydroxy-retinoic acid (4-OH-RA; CAS: 66592-72-1), 4-oxo-retinoic acid (4-oxo-RA; CAS: 38030-57-8), and the internal standard (IS) 4-oxo-retinoic acid-(9-methyl)-*d*_3_ (4-oxo-RA-*d*_3_; CAS: 1346606-26-5) were purchased from Toronto Research Chemicals. 2,6-Di-*tert*-butyl-4-methylphenol (BHT) was supplied by Acros Organics (Geel, Belgium). Sodium pyruvate was purchased from Alfa Aesar (Haverhill, MA). Components of the NADPH-regenerating mix, glucose-6-phosphate, glucose-6-phosphate dehydrogenase, and β-nicotinamide adenine dinucleotide phosphate (NADP+) were purchased from Sigma-Aldrich (St. Louis, MO). All other chemicals and solvents (reagent or analytical grade) were acquired from standard suppliers.

The CYP26A1LR and CYP26B1LR bactosomes (catalog numbers: CYP070 and CYP071, respectively), along with the CYP2C8BR and CYP3A4BR bactosomes (catalog numbers CYP/EZ049 and CYP/EZ005, respectively), were purchased from Cypex Ltd. (Dundee, Scotland, United Kingdom), where the recombinant enzyme was coexpressed with the human cytochrome P450 reductase in *E. coli* and then supplemented with the human cytochrome *b*_5_. CYP26C1 bactosomes were not commercially available for screening. Pooled female primary human hepatocytes (femPHHs; catalog number: KS00039; *n* = 10; ages 16–40), along with associated media, were custom-ordered from XenoTech, LLC/BioIVT (Kansas City, KS).

### Cell Culture and Handling

The femPHHs were thawed using the CryostaX hepatocyte thawing kit (catalog number: K8000) and plated at the recommended seeding densities with the CryostaX hepatocyte plating medium (catalog number: K8200) supplemented with OptiMatrix (catalog number: 8650; 0.25 mg/mL) (XenoTech, LLC/BioIVT; Kansas City, KS). The femPHHs were maintained in a complete culture medium consisting of XenoTech, LLC/BioIVT CryostaX Hepatocyte Culture Media (K8300) supplemented with penicillin/streptomycin (Pen/Strep; PS100) (Kansas City, KS). Cell cultures were incubated at 37 °C with 5% CO_2_. Experimental exposures were conducted 24 h following initial thawing and plating protocols. Cytotoxicity was assessed morphologically and via the LDH-Glo cytotoxicity-assay kit (catalog no. J2380) from Promega (Madison, WI).

### Recombinant CYP *In Vitro at*RA Oxidation

Protein concentration and reaction time were optimized for each CYP to ensure that incubations would be conducted under a linear initial velocity. Incubations were performed with recombinant CYP26 (2.5 pmol/mL CYP26A1LR and CYP26B1LR), CYP2C8, or CYP3A4 (10 pmol/mL), in the presence of *at*RA substrate (3 μM) in 0.1 M potassium phosphate buffer (pH 7.4), 3.3 mM magnesium chloride, 1 mM sodium pyruvate, and 0.02% BHT.^12^ Substrate concentrations were optimized to remain within the linear range of metabolite detection and quantification by our instrument. While *at*RA (3 μM) surpasses the nanomolar *K*_m_ for CYP26 metabolism (*K*_m_ = 50.1 and 18.1 nM for CYP26A1 and -B1 4-OH-RA formation, respectively), potentially lowering inhibitory potency, it remains within that of CYP2C8 (*K*_m_ = 6 μM in microsomes containing cDNA-derived CYP2C8) and CYP3A4 (*K*_m_ = ∼5 μM for 4-OH-RA formation).^30,31,48^ Additionally, these substrate concentrations were consistent with that implemented in previous studies, including those measuring recombinant CYP26 activity.^29–32^ Following a 3 min pre-equilibration step, reactions were initiated by the addition of an NADPH-regenerating mix consisting of NADP^+^ (1 mM), glucose-6-phosphate (10 mM), and glucose-6-phosphate dehydrogenase (2 IU/mL). All reactions were conducted in triplicate for 5 min at 37 °C under gentle agitation in the dark. Final reaction volume reached 200 μL. Dimethyl sulfoxide (DMSO) was used as the solvent control and did not exceed 0.2% final concentration across reactions to minimize nonspecific CYP interactions. Talarozole (TAL; 2 μM) was utilized as the positive control for inhibition. Reactions were stopped by the addition of 200 μL of ice-cold acetonitrile (ACN) containing 4-oxo-RA-*d*_3_ (0.045 μg/mL), and 0.02% BHT. Precipitated proteins were collected by centrifugation for 20 min at 2000 × *g* at 4 °C, and resulting supernatants were transferred to amber high-performance liquid chromatography (HPLC) vials and kept under inert gas until LC-MS/MS analysis of 4-OH and 4-oxo-RA metabolite formation.

### PFAS *In Vitro* Inhibition Screening Assays for the Recombinant CYP26A1 and CYP26B1 Enzyme

A total of 13 PFAS were screened for their potential capacity to inhibit CYP26A1 and CYP26B1 oxidation of *at*RA (Table 1). The experimental setup was based on the CYP3A7 screening assays previously described by Hvizdak et al., 2023.^12^ Six long-chain (PFOA, PFOS, PFNA, PFDA, PFDS, and PFUnDA) and seven short-chain (FBSA, PFBS, PFPeA, GenX, FHxSA, PFHxS, and 4:2 FTS) PFAS stock solutions (5 and 50 mM) were prepared fresh daily in DMSO along with TAL (1 mM). PFAS were combined with the CYP26 reaction mixture described above to yield 10 and 100 μM PFAS final concentrations, respectively. DMSO did not exceed 0.2% final concentration across reactions to minimize nonspecific CYP interactions. Additional incubations without the NADPH-regenerating mix were done in parallel as negative controls. Samples were quantified for 4-OH and 4-oxo-RA metabolite formation compared with the solvent control via LC-MS/MS.

### PFDA IC_50_ Inhibition Assay for CYP26A1, CYP2C8, and CYP3A4 *at*RA Oxidation

PFDA was tested for inhibition of *at*RA oxidation in CYP26A1, and secondary retinoic acid hydroxylase enzymes CYP2C8 and CYP3A4 were tested to obtain half-maximal inhibitory concentrations (IC_50_s) for each enzyme. The IC_50_ assay was performed by utilizing the same experimental conditions outlined above, with the following modifications: PFDA stock and working solutions were prepared fresh daily in DMSO at 5–80 mM (CYP26A1) or 2.5–80 mM (CYP2C8 and CYP3A4) to yield 10–160 μM (CYP26A1) or 5–160 μM (CYP2C8 and CYP3A4) final concentrations. Incubations were performed with CYP26A1LR (2.5 pmol/mL), CYP2C8BR (10 pmol/mL), or CYP3A4BR bactosome (10 pmol/mL) reaction mixtures in the presence of *at*RA substrate (3 μM).^12,49^ Samples were quantified for the formation of the 4-OH and 4-oxo-RA metabolites via LC-MS/MS.

### *In Silico* Docking of *at*RA, 4-OH-RA, and PFDA to the CYP26A1 AlphaFold Homology Model

Given that the human CYP26A1 crystal structure has never been experimentally determined, a suitable homology model was obtained from the AlphaFold database.^50^ The human CYP26A1 predicted amino acid backbone structure (AF-O43174-F1-v4; average p.LDDT: 89.93) was processed in UCSF Chimera (version 1.19).^50,51^ The heme irons on the experimental structures of human neonatal CYP3A7 (PDB: 8GK3) and CYP3A4 (PDB: 1W0E) were utilized as templates for manual insertion of the heme iron into the CYP26A1 homology model. CASTp 3.0 was utilized to depict and measure solvent-accessible sites on the CYP26A1 homology structure.^52^ Receptor ions and water molecules were removed, and polar hydrogens were added using MGL AutoDock Tools (version 1.5.7) (UCSD Molecular Graphics Lab and The Scripps Research Institute) to prepare for ligand docking.^53^ Molecular docking studies of 3D ligand structures obtained from PubChem (https://pubchem.ncbi.nlm.nih.gov/) with the CYP26A1 homology model were performed using AutoDock Vina (version 1.1.2) software for Linux.^54^ Coordinates for the receptor docking grid search space were established in the active site with the following parameters: grid box center: *x*-center = −1.505, *y*-center = −2.806, *z*-center = 0.978, and total number of grid points in each direction: *x*-dimension = *y*-dimension = *z*-dimension = 40. Ubuntu Text Editor was used to prepare a configuration docking script with the exhaustiveness search parameter set to 48. Output files were analyzed using the ViewDock feature in UCSF Chimera.^51^ Docking scores (kcal/mol), predicted residue contacts, and associated distances (Å) for interactions between carbon C4 on the β-ionone ring (*at*RA and 4-OH-RA) or ω carbon (PFDA) and CYP26A1 heme iron were recorded (Table 2). The (4*R*)-OH-RA enantiomer was utilized for docking, given its availability on the PubChem database, and its relative contributions to formation of the 4-oxo-RA metabolite compared to (4*S*).^30^ Relevant docking poses were selected based on energetic favorability and proximity to the heme iron.

**Table 2:** Predicted Parameters and Contacts for CYP26A1 Molecular Docking of Retinoids and PFDA^a^

compound	docking score (kcal/mol)	heme distance (Å)	residue contacts
atRA	–9.0	4.521	E296, G300, T304, P478, V370
(4*R*)-OH-RA	–9.0	5.070	E296, G300, T304, P478, V370
PFDA	–8.1	6.843	P478, V370

a Docking scores (kcal/mol), distances to the heme iron (Å), and predicted residue contacts for each ligand and the CYP26A1 homology model following docking studies. Abbr.: *at*RA (all-*trans*-retinoic acid); 4*R*-OH-RA (4*R*-hydroxy-retinoic acid); PFDA (perfluorodecanoic acid).

### *at*RA and PFDA Dosing in femPHHs

Pooled femPHHs (*n* = 10; ages 16–40) were utilized to assess dose-dependent effects of PFDA on *at*RA metabolism and transcriptomic signaling in the maternal liver. Cells were seeded at 1.0 × 10^6^ cells/mL in 24-well plates precoated with Type I Collagen purchased from XenoTech, LLC/BioIVT (Kansas City, KS). Working stocks of the substrate (*at*RA), inhibitor cocktail TAL and KTC, and PFDA were dissolved in DMSO and prepared fresh daily. Stocks were further diluted in complete culture media (CryostaX Hepatocyte Culture Media (K8300) supplemented with Pen/Strep (PS100)) obtained from XenoTech, LLC/BioIVT (Kansas City, KS). To mimic prenatal retinoid concentrations on the maternal axis, femPHHs were conditioned with *at*RA for 48 h at average plasma levels during pregnancy (5 nM), before our activity assay.^24,26^ During this 48 h period, femPHHs were semi-statically dosed in triplicate every 24 h with DMSO (VC), *at*RA (AC; 5 nM), or *at*RA (5 nM) plus our test compounds: PFDA (1, 25, 50, 75, and 100 μM) or the inhibitor cocktail (TAL/KTC; 5 and 20 μM, respectively) (0.2% *v/v* final DMSO). *at*RA stocks were prepared in the dark, and dosing procedures were carried out away from direct light.

### Cytotoxicity Assay for femPHHs

After the 48 h semi-static exposures, femPHHs were assessed visually for their morphology using an Olympus CKX53 microscope and imaged with an Olympus EP50 digital camera (Tokyo, Japan) at 20× magnification. The LDH-Glo Cytotoxicity assay (Promega; Madison, WI) kit was utilized to measure lactate dehydrogenase (LDH) release according to the manufacturer’s instructions. Before our *at*RA activity assay, supernatant aliquots from each well were diluted 100-fold in LDH storage buffer (200 mM Tris-HCl (pH 7.3), 10% glycerol, 1% bovine serum albumin (BSA)) and assayed according to the manufacturer’s instructions. An LDH standard (4 mU/mL) was prepared by using kit components. Technical duplicates of each biological replicate supernatant sample were averaged and corrected for background luminescence derived from the complete culture medium. Relative cytotoxicity for each sample was determined against the vehicle control (VC, DMSO).

### PFDA Inhibition of *at*RA Metabolism in femPHHs

Following the 48 h semi-static exposures, femPHHs were washed with phosphate-buffered saline (PBS) and treatments with DMSO or test compounds (PFDA or TAL/KTC) were repeated as previously described. All wells conditioned with *at*RA were spiked with the compound at 3 μM for 4 h and incubated at 37 °C, 5% CO_2_. DMSO reached a final concentration of 0.25% (*v/v*) for the *at*RA activity assay. Following the 4 h incubation, supernatant aliquots (200 μL) were collected and added to the same volume of ACN containing 4-oxo-RA-*d*_3_ (0.045 μg/mL) and 0.02% BHT. Stopped femPHH samples were centrifuged, and supernatants were transferred to amber HPLC vials and stored under inert gas until analysis. Samples were assessed for the relative quantification of the 4-OH and 4-oxo-RA metabolites against the *at*RA control (AC) via LC-MS/MS. 4-OH and 4-oxo-RA metabolites in the vehicle control (VC) were additionally quantified for reference of the background retinoic acid and metabolites in our model.

### PFDA Effects on *at*RA Signaling in femPHHs

Following the *at*RA activity assay, femPHHs were washed with PBS, and total ribonucleic acid (RNA) was extracted from each well with the Qiagen RNeasy mini kit (Hilden, Germany) per the manufacturer’s instructions. The end-product RNA was eluted in nuclease-free water and assessed for purity at absorbances of 260/280 nm via the NanoQuant plate with the Tecan Infinite M Plex plate reader (Männedorf, Switzerland). Triplicate RNA samples (∼1 μg) belonging to VC, AC, and those dosed with both *at*RA and PFDA at concentrations below and above the inhibition threshold achieved in our *at*RA activity assay (50 and 75 μM) were submitted to Novogene Corporation, Inc. (Sacramento, CA) for RNA sequencing.

Briefly, messenger RNA (mRNA) was purified from total RNA using poly-T oligo-attached magnetic beads. A standard mRNA library preparation kit was used. Libraries were sequenced on an Illumina NovaSeq X Plus Series. An average of 50 million 150-bp, paired-end reads were obtained from samples sent. Reads were aligned to the GRCh38/Hg38 transcriptome using HISAT2 (version 2.2.1). Differentially expressed genes (DEGs) were quantified by using the DESeq2 R package (version 1.42.0). Kyoto Encyclopedia of Genes and Genomes (KEGG) analysis of differential genes was performed by R package clusterProfiler (version 4.8.1).^55^

### Analytical Method for *at*RA Oxidation

Formation of the 4-OH and 4-oxo-RA metabolites by recombinant enzymes and femPHHs was determined by LC-MS/MS analysis on a Waters Acquity ultra-performance liquid chromatography (UPLC) system interfaced by electrospray ionization (ESI) with a Waters Xevo TQ-S micro tandem quadrupole mass spectrometer. The following source conditions were applied: 2.0 kV for the capillary voltage, 150 °C for the source temperature, 500 °C for the desolvation temperature, and 900 L/h for the desolvation gas flow. A multiple reaction monitoring (MRM) scan type in ESI negative mode was utilized with the following transitions (including collision energies, CE, and cone voltages, CV): 299 > 255 (CE = 14 V, CV = 40 V) for *at*RA, 315 > 253 (CE = 14 V, CV = 45 V) for 4-OH-RA, 313 > 254 (CE = 15 V, CV = 42 V) for 4-oxo-RA, and 316 > 272 (CE = 12 V, CV = 45 V) for 4-oxo-RA-*d*_3_ (IS). Metabolites were separated on a Waters BEH C18 column (1.7 μm, 2.1 mm × 50 mm) in water and ACN containing 5 mM ammonium acetate at 0.4 mL/min with the following gradient: 40% organic held for 0.5 min, increased to 98% over 4 min, and held at 98% for 1 min. The MS peaks were integrated using QuanLynx software (version 4.2, Waters Corp., Milford, MA), and the analyte/internal standard peak area ratios for 4-OH and 4-oxo-RA were determined for the solvent control and used as a reference for 100% activity to calculate the percent remaining activity in the treated samples.

### Statistical Analysis

All experiments were performed in triplicate. The GraphPad Prism 10 software for Windows 64-bit (version 10.4.2) was used for data processing, graph generation, and descriptive statistics (San Diego, CA). Statistical significance was calculated using either two-way analysis of variance (ANOVA; enzymatic assays), or one-way ANOVA (femPHH assays) with Dunnett’s *post hoc* test, and is indicated as follows: * *p* < 0.05, ** *p* < 0.01, and *** *p* < 0.001. Data are represented as mean ± standard deviation (SD). The IC_50_ values and coefficients of determination for PFDA inhibition of recombinant enzyme *at*RA oxidation were calculated by nonlinear regression of the dose–response curve using the log­(inhibitor) vs normalized response-variable slope function in GraphPad Prism.

Statistical analysis of RNA-sequencing data was performed by Novogene Corporation, Inc. (Sacramento, CA). The resulting *p*-values calculated from DEGs were adjusted using the Benjamini–Hochberg (BH) correction for the false discovery rate (FDR). The threshold for significant differential expression is set to “|log_2_(fold change) (log_2_(FC))| ≥ 1.0 and adjusted *p*-value (*p*_adj_) ≤ 0.05” for femPHHs. Data from specific DEGs were processed and visualized utilizing heatmaps displaying the log_2_(FC) values indicated by color. Log_2_(FC) values with *p*_adj_ >0.05 are denoted with a dot ("•"). The top 10 enriched up- and downregulated KEGG pathways were plotted as a function of −log_10_(*p*_adj_) on the horizontal axis, with point size and labels indicating the number of genes annotated to a specific KEGG pathway. KEGG pathways with *p*_adj_ < 0.05 were deemed significant enrichment.

## Results

### PFDA Inhibition of Recombinant CYP26A1 *at*RA Oxidation

The molecular similarity shared between *at*RA and the PFAS compounds with their hydrophobic carbon chain and hydrophilic functional headgroup led us to test a mix of long- and short-chain PFAS for their potential to inhibit CYP26 enzymes. Of the 13 PFAS screened, PFDA was found to be the strongest inhibitor of recombinant CYP26A1 oxidation for both the 4-OH and 4-oxo-RA metabolites (Figure 2). For PFDA, CYP26A1 4-OH-RA formation (percent of control ± SD) remained at 93.9% ± 0.80 (ns, not significant) of the solvent control at 10 μM but decreased to 5.37% ± 0.39 (*p* < 0.001) at 100 μM (Figure 2A). Similarly, CYP26A1 4-oxo-RA formation was 92.6% ± 2.5 (ns) of the solvent control at 10 μM and only 6.97% ± 1.0 (*p* < 0.001) at 100 μM PFDA (Figure 2B). PFDA was the only PFAS that reached over 50% inhibition for the formation of both *at*RA oxidative metabolites. PFUnDA, another long-chain PFAS, had a limited effect on CYP26A1 *at*RA metabolism formation at 10 μM but demonstrated significant loss of 4-OH-RA formation resulting in 57.4% ± 1.3 (*p* < 0.001) remaining activity at 100 μM (Figure 2A). While none of the short-chain PFAS achieved over 50% inhibition (Figure 2C,D), FHxSA significantly inhibited CYP26A1 4-OH-RA metabolite formation with 67.4% ± 3.5 (*p* < 0.001) and 66.6% ± 2.6 (*p* < 0.001) remaining activity at 10 and 100 μM, respectively (Figure 2C). None of the PFAS demonstrated significant inhibition of CYP26B1 oxidation to 4-OH and 4-oxo-RA above 50% at either screening concentration (Supporting Information, Figure S1).

**Figure 2:**
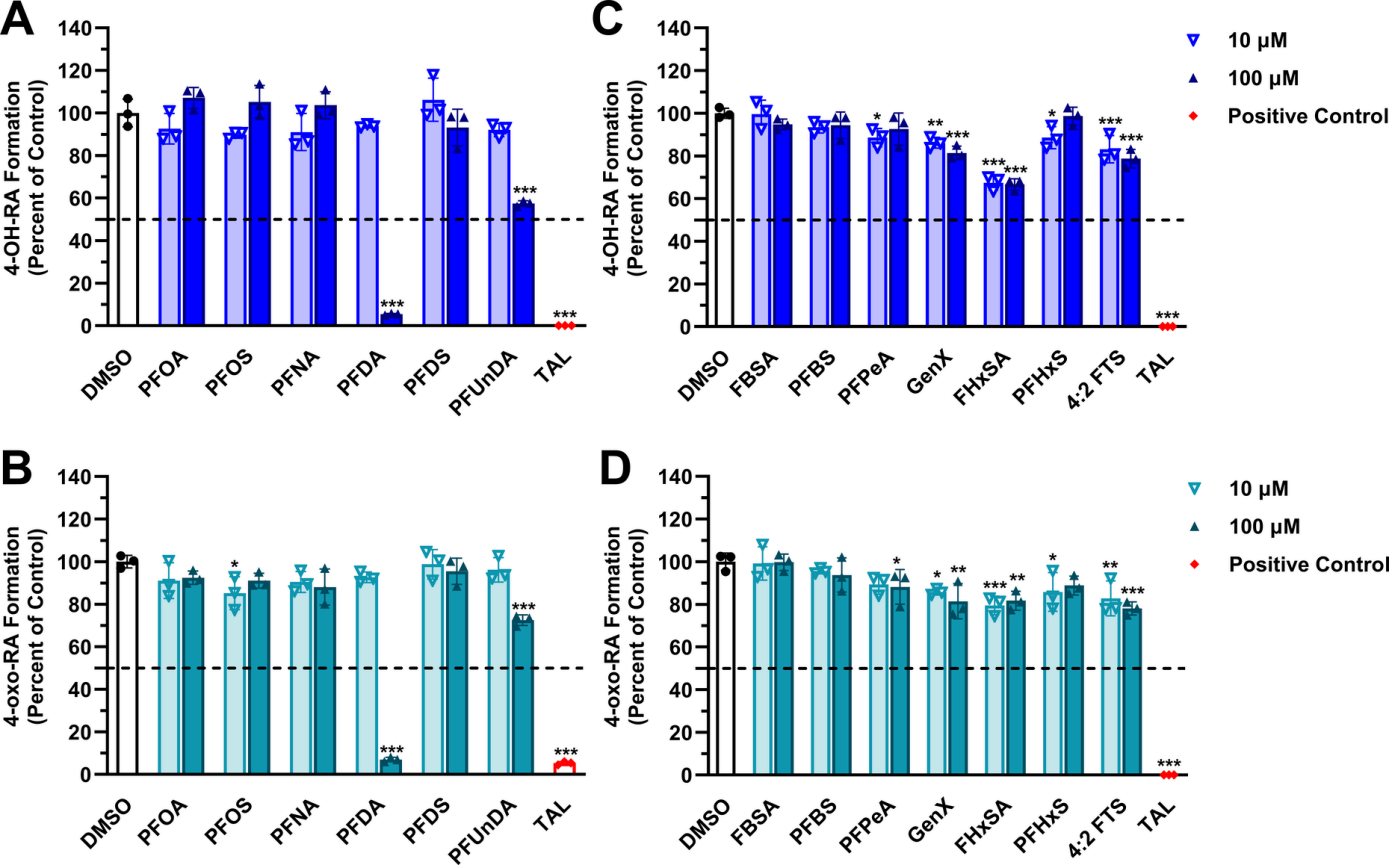
PFAS inhibition screening of recombinant CYP26A1 oxidation of *at*RA. Triplicate values of CYP26A1 4-OH-RA (A, B) and 4-oxo-RA (C, D) as a percent of control following incubation with long-chain (PFOA, PFOS, PFNA, PFDA, PFDS, and PFUnDA; A, C), and short-chain (FBSA, PFBS, PFPeA, GenX, FHxSA, PFHxS, and 4:2 FTS; B, D) PFAS. Talarozole (TAL; 2 μM) was utilized as the positive control for inhibition. Data are represented as mean ± SD. Statistical significance against the solvent control is indicated by * *p* < 0.05, ** *p* < 0.01, and *** *p* < 0.001; two-way ANOVA and Dunnett’s *post hoc* test.

The nearly complete inhibition of CYP26A1 by PFDA at 100 μM led us to further investigate the potency of this PFAS by measuring its IC_50_’s. The IC_50_ values were determined for the 4-OH and 4-oxo-RA metabolites (Figure 3): 49.5 μM (*R* ^2^ = 0.983) and 51.3 μM (*R* ^2^ = 0.988), respectively. These values were consistent with the results obtained from our PFAS screening assay.

**Figure 3:**
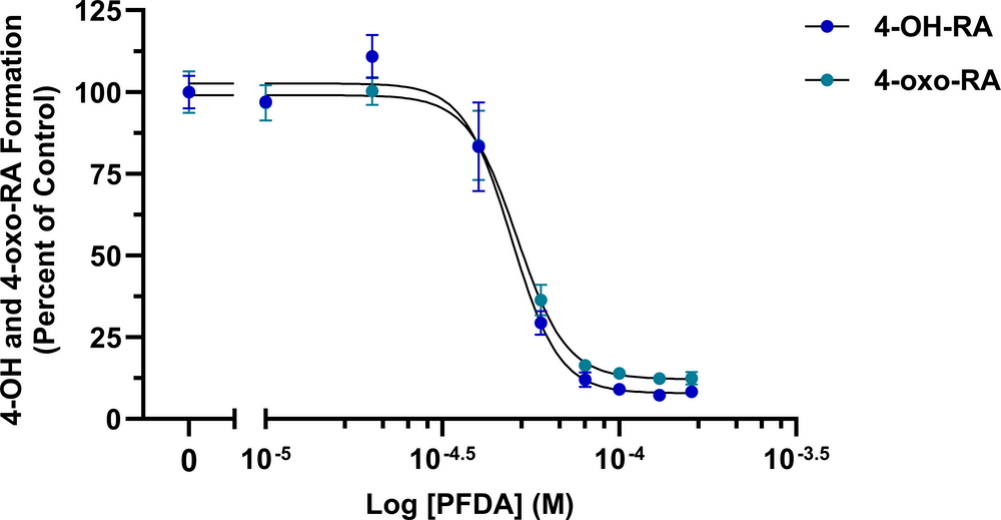
IC_50_ of PFDA inhibition of recombinant CYP26A1 formation of 4-OH and 4-oxo-RA metabolites. CYP26A1 remaining activity is plotted against the log of increasing concentrations of PFDA with IC_50_ = 49.5 μM (*R* ^2^ = 0.983) and IC_50_ = 51.3 μM (*R* ^2^ = 0.988) for 4-OH and 4-oxo-RA, respectively. Triplicate data are represented as mean ± SD. IC_50_ values and coefficients of determination were calculated via nonlinear regression of the dose–response curve using the log­(inhibitor) vs normalized response-variable slope function in GraphPad Prism (version 10.4.2).

### CYP26A1 Homology Model

A CYP26A1 molecular homology model was developed utilizing the AlphaFold structure (AF-Q9PUB4-F1).^50^ The heme prosthetic group was manually inserted into the binding pocket by utilizing UCSF Chimera and the CYP3A7 and CYP3A4 prosthetic heme irons as templates. Upon alignment using the UCSF Chimera MatchMaker function, the CYP26A1 heme iron is positioned 0.242 Å below and 0.749 Å above the CYP3A7 and CYP3A4 heme irons, respectively.^51^ The novel structure yielded typical hallmarks of CYP fold and architecture, with residues (50 total: M1 to G50) of the N-terminus being removed for visualization purposes following our docking studies (Figure 4). CASTp 3.0 defined an active-site volume of 3244 Å^3^ and an area of 2361 Å^2^ on the human AlphaFold-based structure.

**Figure 4:**
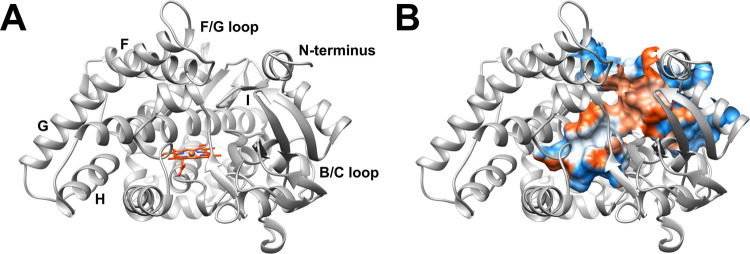
CYP26A1 molecular homology model. (A) Ribbon representation of the CYP26A1 predicted protein structure from AlphaFold (AF-Q9PUB4-F1; dark gray) with identified secondary structures labeled (N-terminus; helices F, G, H, and I; B/C and F/G loops) and depicting the heme prosthetic group manually inserted (orange-red) via UCSF Chimera (version 1.19). (B) CYP26A1 CASTpFold-defined solvent-accessible site, with residues colored according to hydrophobicity (red = more hydrophobic, blue = more hydrophilic, white = neutral). Specific residues belonging to the N-terminus (50 total: M1 to G50) were removed for clarity in visualization.

### Molecular Docking of Retinoids and PFDA to the CYP26A1 Homology Model

To compare the molecular interactions of retinoids and the PFAS compound PFDA within CYP26A1, molecular docking was used with our CYP26A1 homology model to better explore how the carbon chain and carboxylic acid moiety of these ligands sit in the CYP active site. Retinoids *at*RA and 4-OH-RA, along with PFDA, were successfully docked to the CYP26A1 homology model, demonstrating significant overlap in orientation and residue contacts within the ligand-binding pocket (Figure 5 and Table 2). Docking revealed energetically-favorable binding poses for each ligand that feature acceptable distances to the CYP26A1 heme prosthetic group from the C4 position on the β-ionone ring (*at*RA and 4-OH-RA) or the ω carbon (PFDA). *at*RA and 4-OH-RA share identical binding energies (Δ*G*) and residue contacts: −9.0 kcal/mol and E296, G300, T304, P478, and V370 (Figure 5A,B). Docking poses of both retinoids demonstrate the importance of the hydrophobic β-ionone ring in positioning and interacting with the heme iron. In addition, the polar carboxylic acid head groups are pointed away from the heme in an elongated fashion along the I-helix, toward P478 and V370. Comparatively, PFDA demonstrated a slightly weaker affinity for the enzyme, with a docking score of −8.1 kcal/mol (Figure 5C). The hydrophobic ω carbon on PFDA was in close proximity to the heme iron, while the carboxylic acid headgroup is positioned toward P478 and V370, in a manner largely resembling retinoids docked to the same model. An overlay of *at*RA and PFDA visualizes the similarities in docking orientation within the CYP26A1 binding pocket, with mutual occlusion between *at*RA and PFDA (Figure 5D). Distances between the C4 position on the β-ionone ring (retinoids) or the ω carbon (PFDA) and the heme iron are as follows: *at*RA = 4.521 Å, 4-OH-RA = 5.070 Å, and PFDA = 6.843 Å (Figure 5).

**Figure 5:**
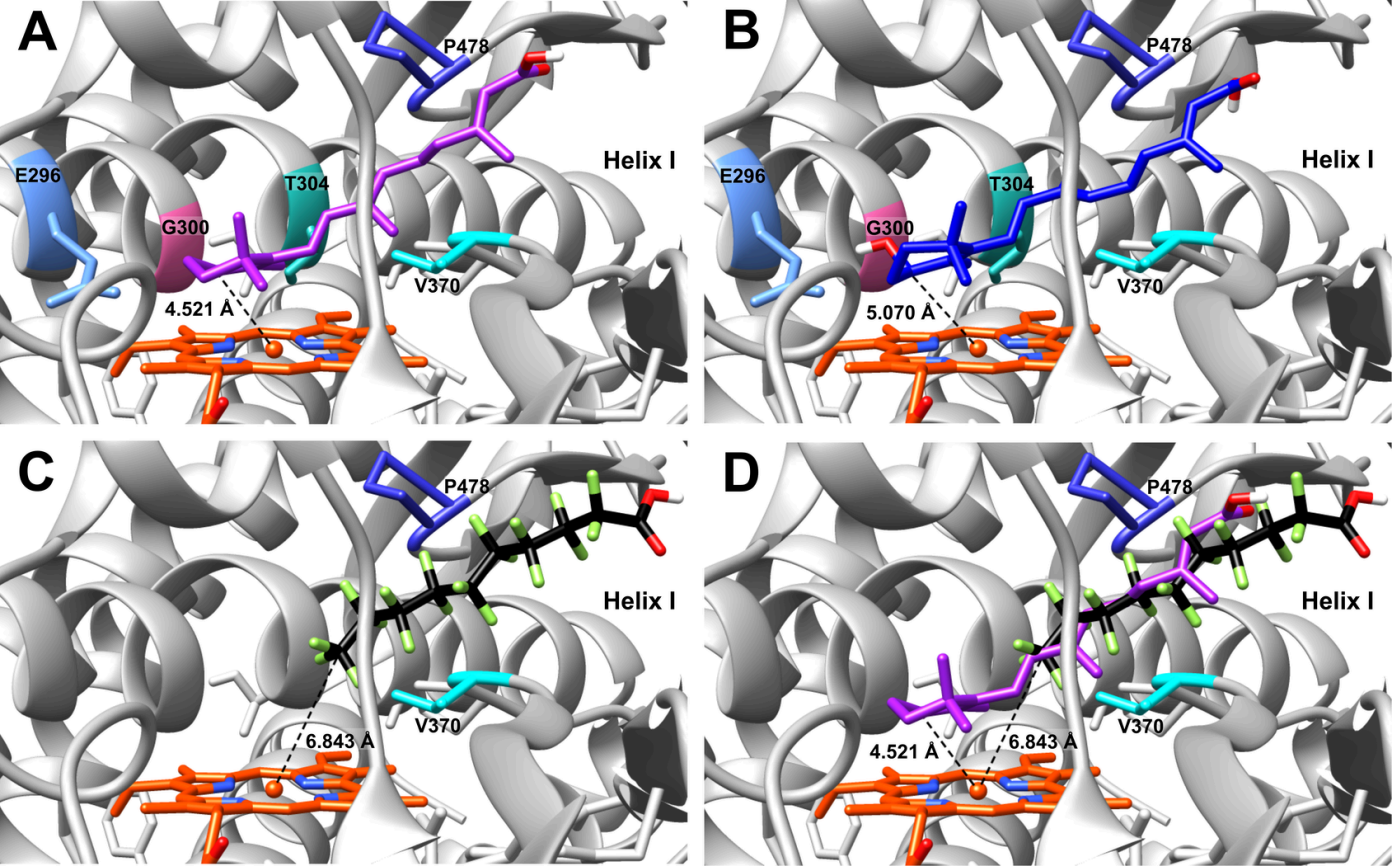
*In silico* docking of retinoids and PFDA with the CYP26A1 homology model. CYP26A1-active site cutaway of the docked structure of *at*RA (A), (4*R*)-OH-RA (B), PFDA (C), and an overlay of *at*RA and PFDA (D) with reference to helix “I”. Predicted residue contacts for each ligand are colored and labeled, with shared residues for *at*RA and PFDA indicated in panel (D) (colors according to UCSF Chimera: E296, cornflower blue; G300, hot pink; T304, light sea green; P478, medium blue; V370, cyan). Distances between the CYP26A1 heme iron and sites of oxidation on retinoids or the PFDA ω carbon are represented by the dashed black line and labeled in Å: *at*RA (A: 4.521 Å), (4*R*)-OH-RA (B: 5.070 Å), PFDA (C: 6.843 Å).

### Cytotoxicity in the femPHH *at*RA Exposure Assay

Based on the results from our recombinant CYP26A1 screening and IC_50_ assays, we endeavored to recapitulate the PFDA inhibition of *at*RA oxidation in an advanced model system. We utilized femPHHs (ages 16–40) to model the retinoid signaling and metabolism pathway in pregnant persons, by dosing for 48 h with mean *at*RA concentrations found in maternal plasma during pregnancy (5 nM), along with PFDA or the TAL/KTC retinoic acid hydroxylase inhibitor cocktail.^24,26^ Before performing the *at*RA activity assay, femPHHs were examined morphologically and imaged, and supernatant was collected to measure cytotoxicity brought on by the semi-static exposure conditions in the *at*RA control (AC, *at*RA; 5 nM), *at*RA (5 nM) plus PFDA (1–100 μM) or the TAL (5 μM)/KTC (20 μM) inhibitor cocktail, against the vehicle control (VC). Our findings indicated that femPHHs were resilient to any mortality induced by basal levels of *at*RA (AC) along with *at*RA plus PFDA (1–75 μM) in our hepatic cell model following the 48 h semi-static exposure period (Figure 6 and Supporting Information, Figure S2). However, significant LDH release was measured for the 100 μM PFDA dosing (percent of VC ± SD: 368% ± 18; *p* < 0.001) and TAL/KTC inhibitor cocktail (320% ± 54; *p* < 0.001). The values were comparable to the LDH standard (4 mU/mL), which emitted an average luminescence signal equivalent to 355% of our VC. Both the femPHHs in the 100 μM PFDA and the inhibitor cocktail groups exhibited morphological hallmarks of cytotoxicity, featuring membrane blebbing and fragmentation of nuclei. However, the 100 μM PFDA group largely maintained adhesion compared to that in the inhibitor cocktail group (Figure S2). Given these results, the metabolic competency of the femPHHs treated with TAL/KTC or 100 μM PFDA at 48 h may be compromised, as these treatments proved cytotoxic in our hepatic cell model.

**Figure 6:**
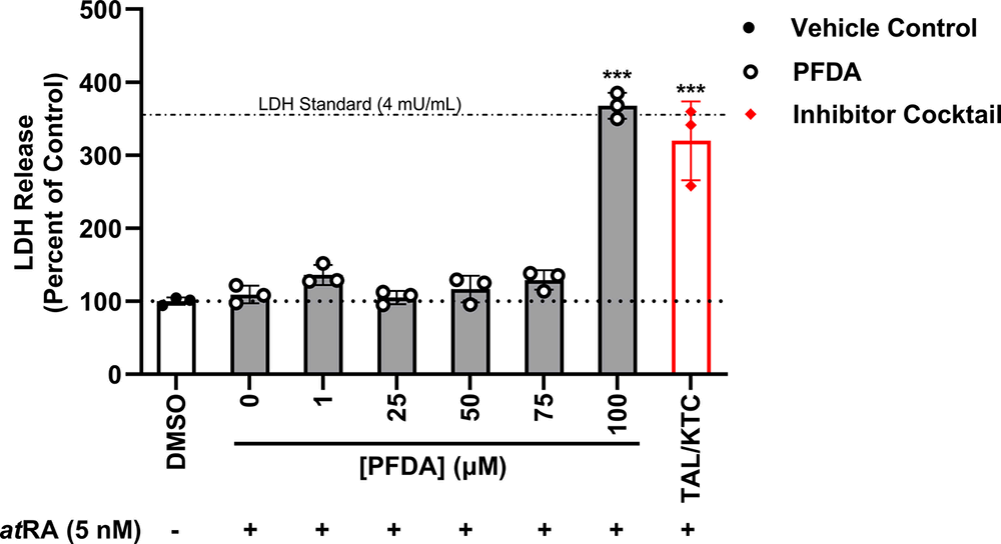
LDH release in femPHHs following 48 h semi-static exposures to PFDA and *at*RA activity conditions. Percent of control (VC) for LDH release in female primary human hepatocytes (femPHHs) is plotted in triplicate against increasing concentrations of PFDA (0–100 μM) and the talarozole/ketoconazole (TAL/KTC; 5 and 20 μM, respectively) inhibitor cocktail. Groups exposed to basal concentrations of *at*RA (5 nM) over the 48 h duration are indicated below the *x*-axis. The LDH standard positive control (4 mU/mL) corresponding to 355% release is designated by the semidashed line. Data are represented as mean ± SD. Statistical significance against the DMSO control is indicated by * *p* < 0.05, ** *p* < 0.01, and *** *p* < 0.001; one-way ANOVA and Dunnett’s *post hoc* test.

### PFDA Perturbs *at*RA Oxidation in femPHHs

After performing our cytotoxicity assay, the treated femPHHs were tested for *at*RA metabolic activity by spiking the cells with 3 μM *at*RA to serve as probe substrate and by incubating them for 4 h to allow for detectable product formation by LC-MS/MS. Formation of 4-OH and 4-oxo-RA in the VC remained at 0.00% (*p* < 0.001), indicative of the lack of background retinoids or contamination in our model. At 1–50 μM PFDA, metabolite formation was not inhibited compared to that of AC (Figure 7). On the contrary, an apparent increase (percent of AC) was observed in *at*RA metabolites at intermediate PFDA concentrations, with average formation (percent of AC ± SD) peaking at 25 μM PFDA for both metabolites: 131% ± 4.3 for 4-OH-RA (Figure 7A) and 117% ± 3.6 for 4-oxo-RA (Figure 7B). However, treatment with 75 μM PFDA significantly diminished metabolism in femPHHs, at 22.5% ± 5.4 and 12.2% ± 3.8 for 4-OH and 4-oxo-RA formation, respectively (*p* < 0.001) (Figure 7A,B). In addition, no formation of *at*RA metabolites was detected in femPHHs treated with 100 μM PFDA or the inhibitor cocktail. Despite the atypical dose–response patterns observed with the femPHH retinoid metabolites, overall *at*RA clearance was significantly impeded by PFDA between 50 and 100 μM (*p* < 0.001). At 75–100 μM PFDA, *at*RA levels exceeded those in the TAL/KTC inhibitor cocktail, reaching 182% AC (75 μM PFDA) (Figure 7C).

**Figure 7:**
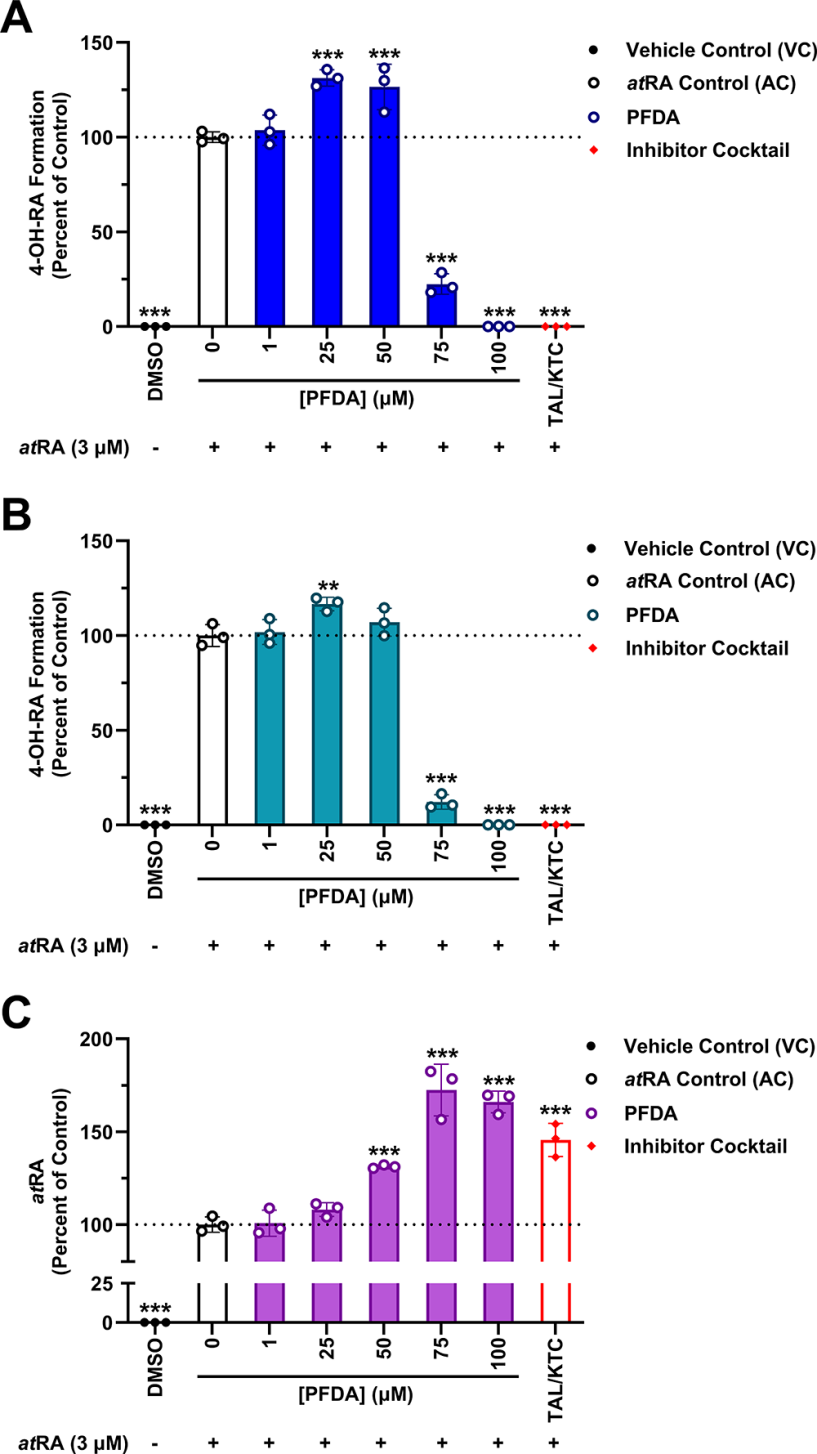
PFDA inhibits femPHH metabolism of *at*RA *in vitro*. Percent of control (*at*RA control, AC; *at*RA, 3 μM; PFDA, 0 μM) for 4-OH-RA (A), 4-oxo-RA (B), and *at*RA (C) quantification is plotted in triplicate against increasing concentrations of PFDA (1–100 μM) and the talarozole/ketoconazole (TAL/KTC; 5 and 20 μM, respectively) inhibitor cocktail. Groups spiked with *at*RA (3 μM) following the 48 h semi-static incubations are indicated below the *x*-axis. Presence of 4-OH-RA, 4-oxo-RA, and *at*RA in the vehicle control (VC, DMSO) was additionally plotted for reference to background retinoids in our model. Data are represented as mean ± SD. Statistical significance against the *at*RA control is indicated by * *p* < 0.05, ** *p* < 0.01, and *** *p* < 0.001; one-way ANOVA and Dunnett’s *post hoc* test.

According to our cytotoxicity data, the 100 μM PFDA and TAL/KTC inhibitor cocktail groups displayed significant LDH release and morphological changes following the 48 h semi-static exposures compared to the VC (Figure 6 and Figure S2). Therefore, femPHHs belonging to these groups may have lacked the metabolic competency to clear *at*RA, unlike those treated with 75 μM PFDA in which we observed a reliable inhibitory response brought on by the test compound. Nevertheless, our cellular *at*RA oxidation data demonstrate that PFDA can significantly decrease *at*RA metabolism (75 μM PFDA) and clearance (50–75 μM PFDA) in femPHHs at noncytotoxic concentrations (Figure 7C).

### PFDA IC_50_ for CYP2C8 and CYP3A4 *at*RA Oxidation

Due to the inhibition of *at*RA oxidation brought on by PFDA in our advanced femPHH model, we performed additional IC_50_s with CYP2C8 and CYP3A4 (Figure 8) to determine the effects of PFDA on secondary retinoic acid hydroxylases (Figure 1) present in our system. Half-maximal inhibitory concentrations for CYP2C8 were 85.4 μM (*R* ^2^ = 0.973) and 68.0 μM (*R* ^2^ = 0.979) for 4-OH and 4-oxo-RA, respectively (Figure 8A). PFDA significantly inhibited CYP3A4 4-OH-RA formation, with an IC_50_ value of 28.4 μM (*R* ^2^ = 0.988) (Figure 8B). For CYP3A4, the formation of 4-oxo-RA was not measured due to the sensitivity limitations inherent in the assay.

**Figure 8:**
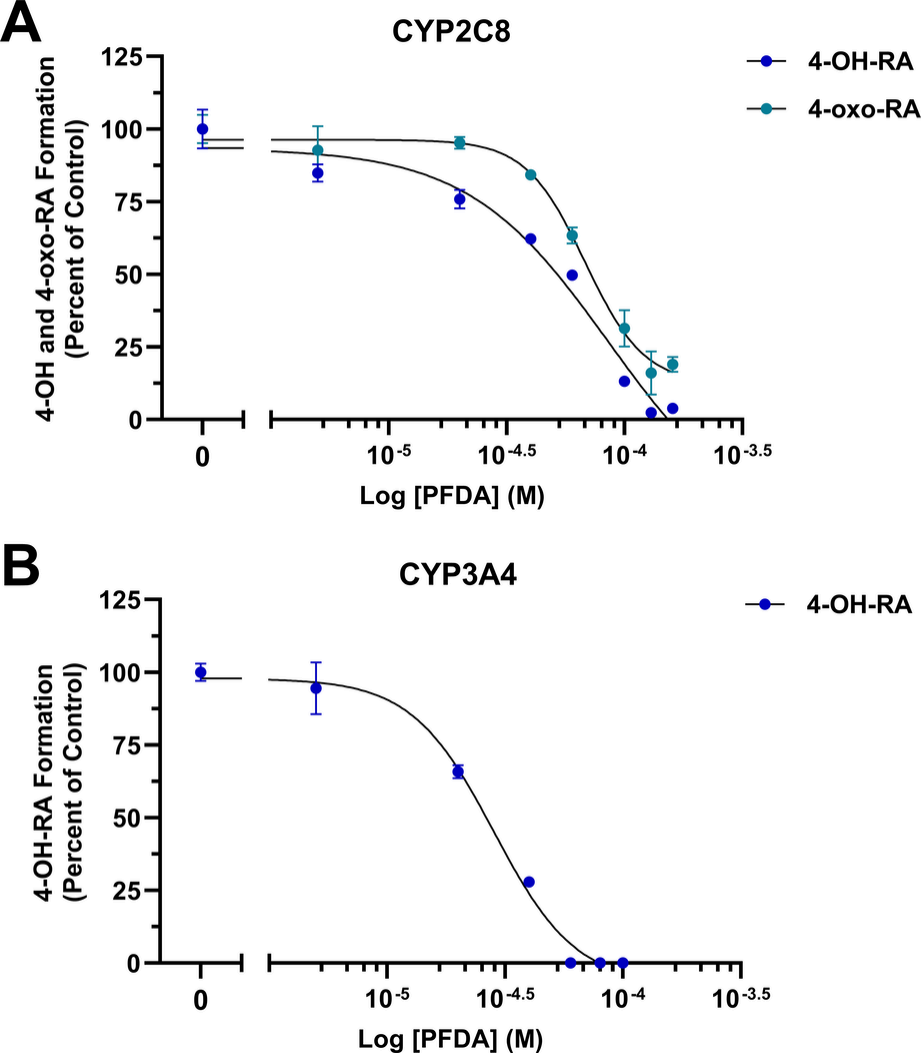
IC_50_ of PFDA inhibition of recombinant hepatic retinoic acid hydroxylases CYP2C8 and CYP3A4. CYP2C8 (A) and CYP3A4 (B) remaining activity is plotted against the log of increasing concentrations of PFDA. For CYP2C8, IC_50_ = 85.4 μM (*R* ^2^ = 0.973) and IC_50_ = 68.0 μM (*R* ^2^ = 0.979) for 4-OH and 4-oxo-RA, respectively. For CYP3A4, IC_50_ = 28.4 μM (*R* ^2^ = 0.988) for 4-OH-RA. The IC_50_ for CYP3A4 formation of 4-oxo-RA is not plotted due to the sensitivity limitations in the assay. Triplicate data are represented as mean ± SD. IC_50_ values and coefficients of determination were calculated via nonlinear regression of the dose–response curve using the log­(inhibitor) vs normalized response-variable slope function in GraphPad Prism (version 10.4.2).

### PFDA Dysregulates the Expression of Genes Involved in Retinol Metabolism and *at*RA Signaling

RNA-sequencing analysis of femPHHs dosed with *at*RA and nonlethal concentrations of PFDA (50 and 75 μM) revealed that retinol metabolism (map00830) was among the top five downregulated KEGG-enriched pathways when controlling for *at*RA (AC) (*p*_adj_ < 0.05) (Figure 9A,B). The drug metabolism cytochrome P450 (map00982) and metabolism of xenobiotics by cytochrome P450 (map00980) were among the second- and fourth-most downregulated KEGG pathways at both concentrations, respectively (*p*_adj_ < 0.05) (Figure 9A,B). Following PFDA exposures, the most downregulated pathway was the complement and coagulation cascades (map04610) (50 and 75 μM PFDA vs AC), and the most upregulated pathways were cell cycle (map04110) and aminoacyl-tRNA biosynthesis (map00970) for 50 μM PFDA vs AC and 75 μM PFDA vs AC, respectively (Figure 9A,B).

**Figure 9:**
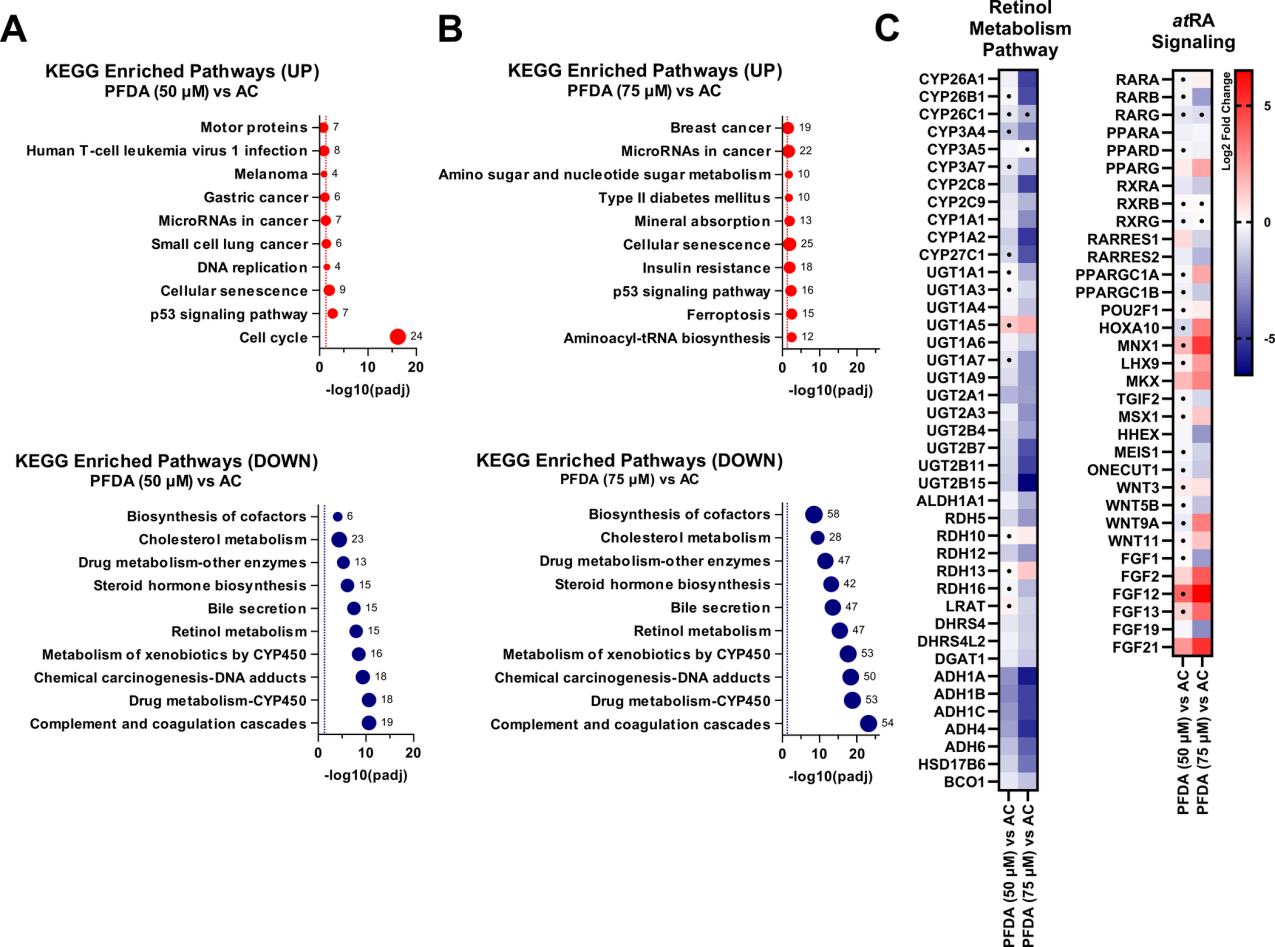
Enriched KEGG pathways and analysis of differentially expressed genes in femPHHs dosed with PFDA. Female primary human hepatocytes (femPHHs) were exposed in triplicate to PFDA-treated hepatocyte culture media. femPHHs were dosed with *at*RA (5 nM) for 48 h, followed by an *at*RA spike (3 μM) and a 4 h incubation. Enriched up- (red) and downregulated (blue) KEGG pathways following 50 and 75 μM PFDA (A and B, respectively) were ranked based on significance scaled as −log_10_(*p*_adj_) on the horizontal axis. Point size and the adjacent labels correspond to the number of genes annotated to a specific KEGG pathway. Statistical significance (*p*_adj_ < 0.05) against the *at*RA control (AC) is indicated by each dashed line. (C) Heatmaps of differentially expressed genes within specific KEGG pathways, including those related to the retinol metabolism pathway and downstream targets of *at*RA signaling. The color scale represents the log_2_(fold change) expression. The threshold of significant differential expression is “|log_2_(fold change)| ≥ 1.0 and adjusted *p*-value (*p*_adj_) ≤ 0.05”. Adjusted *p*-values were determined using the Benjamini–Hochberg (BH) correction for the false discovery rate (FDR). Log_2_(fold change) values with *p*_adj_ >0.05 are denoted with a dot ("•"). Statistical analysis was performed by Novogene Corporation, Inc. (Sacramento, CA).

As expected, retinol metabolism (map00980) was significantly upregulated in the AC compared to the VC (*p*_adj_ < 0.05) (Supporting Information, Figure S3A). This upregulation was reflected in the expression of *CYP26A1*, *-B1*, and *-C1* in the AC, which exhibited log_2_(FC) values of 12.1, 10.7, and 4.15 (*p*_adj_ ≤ 0.05), respectively. The expression of other retinoic acid hydroxylases, including genes coding for members of the CYP2C and CYP3A family, had |log_2_(FC)| < 1.0 (*p*_adj_ ≤ 0.05). Based on these results, the 48 h semi-static exposures to *at*RA in the femPHH model likely primed for CYP26-related metabolic activity.

Log_2_(FC) values of −4.92 and −4.59 (*p*_adj_ ≤ 0.05) were noted for *CYP26A1* and *-B1*, respectively, in the femPHHs dosed with 75 μM PFDA when corrected for *at*RA (AC) (Figure 9C). In addition, *CYP2C8* was significantly downregulated by −1.11 (50 μM PFDA) and −4.91 (75 μM PFDA) along with *CYP3A4* at −3.22 (75 μM PFDA) compared to AC (*p*_adj_ ≤ 0.05) (Figure 9C). *CYP1A2*, UDP-glucuronosyltransferase 2B15 (*UGT2B15*), and alcohol dehydrogenase 1A (*ADH1A*) were also among the most dysregulated genes in the retinol metabolism pathway, featuring a log_2_(FC) of −5.14, −6.57, and −5.84 (75 μM PFDA vs AC; *p*_adj_ ≤ 0.05), respectively (Figure 9C).

Genes regulated by *at*RA signaling, including RARβ (*RARB*), RXRα (*RXRA*), and peroxisome proliferator-activated receptor gamma (PPARγ) (*PPARG*), exhibited log_2_(FC) values of −2.50, −1.37, and 2.29, respectively (75 μM PFDA vs AC; *p*_adj_ ≤ 0.05) (Figure 9C). Wnt family member 5B (*WNT5B*) and 9A (*WNT9A*), both involved in craniofacial morphogenesis and skeletal development, displayed a log_2_(FC) of −1.49 and 3.25, respectively (75 μM PFDA vs AC; *p*_adj_ ≤ 0.05) (Figure 9C).^56–58^ Homeobox A10 (*HOXA10*) was induced (embryo implantation and uterine development; log_2_(FC) = 3.26). Altered *HOXA10* expression can negatively impact fertility in female adults.^59^ Other notable hits include motor neuron and pancreas homeobox 1 (*MNX1*) (motor neuron, spinal cord, and pancreatic development; log_2_(FC) = 4.99), and hematopoietically expressed homeobox (HHEX) (liver development and bile duct morphogenesis; log_2_(FC) = −2.69) (75 μM PFDA vs AC; *p*_adj_ ≤ 0.05) (Figure 9C).^60–62^ It has been found that inhibition of *at*RA signaling results in a downregulation of HHEX expression during human embryonic stem cell (hESC) differentiation to pancreatic cells.^63^

Retinoic acid is known to regulate the expression of fibroblast growth factor (FGF) genes during critical stages of embryonic development.^64^ Retinoic acid and FGF signaling pathways have also been reported to antagonize one another throughout body axis extension and limb formation.^65,66^ *FGF2* (angiogenesis), *FGF12* (neuronal development), and *FGF21* (fetal growth and glucose/lipid metabolism) featured a log_2_(FC) of 4.12, 6.51, and 5.11, respectively (75 μM PFDA vs AC; *p*_adj_ ≤ 0.05) (Figure 9C).^67–70^ In the adult liver, *FGF21* is strongly correlated with the obese state, and its upregulation may be indicative of a compensatory response to elevated adiposity and metabolic stress.^71^ Upon comparison of the AC and VC, *at*RA appeared to significantly induce *RARB* (log_2_(FC) = 6.01) and *FGF19* (bile acid synthesis and glucose/lipid metabolism) (log_2_(FC) = 9.10) when compared to other genes impacted by *at*RA signaling (*p*_adj_ ≤ 0.05) (Supporting Information, Figure S3C).^72^ The indexed gene list is available in the Supporting Information (Table S1).

## Discussion

There is a growing body of evidence attributing various phenotypic effects in chordates developmentally exposed to PFAS. These primarily consist of craniofacial abnormalities, including dysmorphology of the head or eyes, an atypical philtrum, shorter palpebral fissure lengths (PFLs), and increased cleft palate occurrence in humans and laboratory animals.^2–5^ In Truong et al., a systematic developmental toxicity screening of 139 PFAS was performed in zebrafish.^3^ Among the PFAS tested, PFDA was the most developmentally toxic, with the lowest benchmark dose corresponding to 10% increased risk (BMD_10_) for morphological defects at 223 nM.^3^ Of the 13 morphological end points measured, PFDA was most strongly associated with craniofacial abnormalities characterized by a malformed, smaller-than-normal, or missing eye, snout, and/or jaw following static exposures (0.015 to 100 μM) at 120 h postfertilization.^3^ Evidence from human epidemiological studies correlates with the experimental results from laboratory animals, implicating PFDA in craniofacial defects. In a subset of the Danish National Birth Cohort (DNBC) encompassing 656 children, PFDA in maternal serum demonstrated the strongest association with shorter PFLs (distance between the medial and lateral canthus (corner of the eye)) in children five years of age, of the six prominent (detected in >90% maternal serum samples) PFAS examined (OR = 2.02; 95% CI: 1.11, 3.70).^2^ A study investigating prenatal eye morphogenesis in mice determined that adult RAR mutants (RARβ2 single null and RARβ2/RARγ2 heterozygous) displayed significantly shorter PFLs compared to the WT, among other defects of the eye.^73^ The results from this study suggest that PFLs in mice are regulated in-part by retinoid signaling and may serve as a biomarker for maternal vitamin A deficiency (VAD) during chordate development.

Based on our findings presented in this study, of the 13 PFAS tested, PFDA demonstrated the highest capacity to inhibit *at*RA oxidation by recombinant CYP26A1 with an IC_50_ of 49.5 μM (*R* ^2^ = 0.983) and 51.3 μM (*R* ^2^ = 0.988) for 4-OH and 4-oxo-RA, respectively (Figures 2 and 3). In fact, PFDA bears the strongest chemical resemblance to the native retinoid substrates of the CYP26 enzyme family. PFDA and retinoic acids share a polar carboxylic acid headgroup. Additionally, the 10-carbon chain length of PFDA mimics that of the retinoic acid conjugated side chain attached at the sixth position of the β-ionone ring, excluding the two methyl substituents (Figure 1). Like PFDA and *at*RA, PFUnDA possesses a carboxylic acid headgroup, potentially driving the partial inhibition of CYP26A1-mediated activity (Figure 2). However, PFUnDA bears 11 carbons in its fluorinated backbone chain, which impacted its capacity to fully inhibit the reaction. On the other hand, despite the 10-carbon chain length found on PFDS, its sulfonic acid headgroup diminished all affinity for the CYP26A1 active site. These data suggest that the polar headgroup, and to a lesser degree the carbon chain length, may be instrumental for ligand recognition in CYP26A1-specific interactions. We additionally observed CYP26A1 inhibition by FHxSA that was neither dose-dependent nor consistent with the expected sequential metabolite profiles for 4-OH and 4-oxo-RA formation (Figure 2C,D). Given the stability of the IS, plausible explanations for this include the compound’s limited solubility in DMSO and potential interference with analyte-specific 4-OH-RA MRM detection. Further testing is needed to validate these possibilities. It is important to note that we did not observe any PFAS inhibition of recombinant CYP26B1 *at*RA metabolism (Supporting Information, Figure S1). This may be because CYP26B1 has a ∼2.7-fold higher affinity (*K*_m_ = 18.8 nM for 4-OH-RA formation) for *at*RA compared to CYP26A1 and therefore may demonstrate higher specificity for ligands entering its active site.^31^

While basal PFDA exposure levels typically occur at or below the low nanomolar range in human tissues, the highest recorded value to date is approximately 204 ng/g in the brain of one individual from Spain (∼413 nM based on average brain density at 1.04 g/mL according to Barber et al.).^40–42,74–80^ In a literature review conducted by Beggs and colleagues, PFAS have been shown to reach low-to-mid micromolar concentrations in human serum, particularly in occupational cohorts and residents of polluted areas across the United States.^81–83^ According to the available human biomonitoring data from comparable cohorts worldwide, PFDA is elevated to the following maximum levels in serum: professional ski waxers (28 ng/mL, ∼54.5 nM; *n* = 13), fishery employees at Tangxun Lake, China (∼60 ng/mL, ∼117 nM; *n* = 39), and residents near a fluorochemical plant in Jiangsu Province, China (68 ng/mL, ∼132 nM; *n* = 132).^76,78,84,85^ While these values fall well below the micromolar IC_50_s measured in this study, it is necessary to recognize that PFDA global biomonitoring remains an ongoing area of research, with the compound relatively underrepresented in the literature compared to PFOA and PFOS.^86^ Furthermore, the estimated biological elimination half-life for PFDA is 12 years in human serum, indicating the possibility for long-term bioaccumulation.^87^ Albumin is the dominant carrier protein of PFDA, facilitating its interactions with bile acid transport mechanisms.^88,89^ As demonstrated in rats and fish species, PFDA can undergo extensive enterohepatic circulation, allowing for continuous delivery to retinoic acid hydroxylases in maternal liver.^90^ Thus, it may be possible for hepatic concentrations to surpass inhibitory thresholds in select individuals, particularly if PFDA accumulates at high levels in the liver. Moreover, due to the low BMD_10_ for morphological defects in zebrafish (223 nM) and associations with craniofacial defects, we posit that its teratogenic index may be lowered by the impacts of PFDA on transcriptomic signaling (including retinoid pathways (Figure 9)) and *at*RA metabolism by fetal-specific retinoic acid hydroxylases, which remains to be studied *in vitro*.^2,3^ It is important to note that even small perturbations of the relative concentrations of *at*RA at critical stages of development, while not embryonically lethal, could lead to some of the severe developmental abnormalities that are observed with PFAS exposure.

We have previously demonstrated the capacity for PFAS to bind to and inhibit another retinoic acid hydroxylase, human neonatal CYP3A7.^12^ While we were unable to obtain purified CYP26A1 protein for binding studies, we can posit that the two share topographical similarities in their active sites, with CYP3A7 being the more promiscuous of the two, given the range of its known ligand interactions.^91^ Instead, we performed molecular docking to compare PFDA and the retinoids *at*RA and (4*R*)-OH-RA, in their potential interactions within the CYP26A1 binding pocket. Given that currently no crystal structure exists for CYP26A1, a homology model was sourced from the AlphaFold database, and the CYP3A7 and CYP3A4 experimental structures were used as templates for manual insertion of the iron-containing heme.^50^ CASTp 3.0 identified a large solvent-accessible site volume of 3244 Å^3^ and area of 2361 Å^2^, into which PFDA could dock to the AlphaFold-based structure (Figure 4B).^52^ This predicted active site is larger than a previously defined CYP26A1 active-site homology model based on the bacterial CYP120 crystal structure (active-site volume: 918 Å^3^) (Figure 4B).^92^ Other CYP26A1 homology models based on both bacterial and human CYPs did not report a defined area or volume of their predicted active sites.^93,94^

In the CYP26A1 homology model, the most energetically favorable binding pose for PFDA featured the ω carbon positioned closest to the heme iron (Figure 5C). Given that productive binding of retinoids relies on coordination with the β-ionone ring (Figure 5A,B), it is not surprising that PFDA docked with its hydrophobic tail pointed toward the CYP26A1 heme (Figure 5C). The PFDA ω carbon was located at 6.843 Å (Δ*G* = −8.1 kcal/mol) from the CYP26A1 heme iron in the homology model (Figure 5C and Table 1), which aligned well with what was previously observed for Type I interactions between PFAS and CYP3A7.^12^ The docking of the retinoids was also consistent with other CYP26A1 homology models, despite the fact that most of these models were built based on high sequence identity to previously crystallized bacterial or human CYPs as templates.^30,93,95^ The distance between the carbon C4 of *at*RA and the CYP26A1 heme iron in the homology model is 4.521 Å (Δ*G* = −9.0 kcal/mol), which is among the closest distances reported for this specific interaction, generally ranging from 4.16 to 5.6 Å (Figure 5A and Table 2).^30,93,95,96^ The orientation of the *at*RA ring, hovering over the heme iron in the CYP26A1 binding pocket, allows for an attack at other productive oxidation sites, including the C16 and C18 positions. Additionally, the β-ionone ring of *at*RA docked on the alpha side favors an attack toward the (4*S*) enantiomer over the (4*R*) derivative (Figure 5A). This corroborates previous reports of the stereoselective metabolism of CYP26A1, indicating a significantly higher formation of (4*S*) compared to (4*R*)-OH-RA.^30^ This “pro-*S*” oxidation pattern has also been documented in other CYP26A1 homology models.^30,92^ Given that (4*R*)-OH-RA is known as the major contributor to 4-oxo-RA production, we docked it to CYP26A1 and observed similar results (Figure 5B). Like its parent compound, (4*R*)-OH-RA docked on the *S*-(alpha) side, and its carbon C4 sits at a distance of 5.070 Å (Δ*G* = −9.0 kcal/mol) to the heme iron (Table 2). Both *at*RA and (4*R*)-OH-RA interacted with the same five active-site residues (E296, G300, T304, P478, and V370) (Figure 5A,B), with E296, G300, P478, and V370 reported as predicted *at*RA residue contacts in other models.^95,96^ In our docking study, the shared residue contacts for *at*RA and PFDA (P478 and V370) may play a role in anchoring the hydrophobic chain above the base of the heme iron (Figure 5C,D). Therefore, they could be instrumental in placing PFDA as an interferent with *at*RA binding in the CYP26A1 active site.

During embryogenesis, the fetus is entirely dependent on maternal circulating retinoids to meet physiological demands, as *at*RA cannot be synthesized *de novo*.^16,17^ *at*RA concentrations in maternal plasma are increased during pregnancy compared to postpartum, peaking midpregnancy, and averaging around 5 nM across all trimesters.^24,26^ We utilized pooled female primary human hepatocytes (femPHHs) of reproductive age (*n* = 10; ages 16–40) to model maternal hepatic *at*RA homeostasis in the presence of increasing concentrations of PFDA and known retinoic acid hydroxylase inhibitors. By conditioning the femPHHs with *at*RA for 48 h coupled with semi-static incubations with the test compounds, we endeavored to physiologically mimic prenatal PFAS exposures in the relative context of *at*RA metabolism and signaling. At 75 μM PFDA, we observed a 77.5% ± 5.4 reduction in 4-OH-RA formation and an 87.8% ± 3.8 reduction in 4-oxo-RA formation compared with the *at*RA control (AC) (Figure 7). Additionally, 4-OH and 4-oxo-RA formation was not detected at the 100 μM PFDA and TAL/KTC inhibitor cocktail treatments (Figure 7).

The significant LDH release and morphological changes documented in the 100 μM PFDA and TAL/KTC inhibitor cocktail groups limit our interpretation of these data points, which showed zero formation of *at*RA metabolites. The cytotoxicity data indicate that these groups may present a lack of metabolic competency and not necessarily a reliable inhibition of *at*RA hydroxylation, in contrast to our 75 μM PFDA data (Figure 6 and Supporting Information, Figure S2). It is important to note that membrane blebbing and nuclei fragmentation were not observed in the 100 μM PFDA group at 24 h (data not shown), suggesting that bioaccumulation resulting from semi-static dosing allowed for intracellular concentrations to reach levels over a relatively narrow effective threshold for PFDA cytotoxicity. Kam et al. demonstrated PFAS cytotoxicity in HepG2 cells to be largely chain-length dependent, with inhibition of mitochondrial activity a major contributing factor to cellular damage after 48 h exposure to 100 μM PFDA.^97^ During our preliminary optimization experiments, 5 μM TAL did not induce hepatotoxicity (data not shown). Therefore, the addition of 20 μM KTC to the inhibitor cocktail likely contributed to the significant LDH release. It is widely accepted that KTC hepatotoxicity is brought on by its *N*-deacetyl ketoconazole intermediate via metabolism by human arylacetamide deacetylase.^98^ While ketoconazole cytotoxicity studies have not been performed in primary human hepatocytes, KTC did not induce significant hepatotoxicity in modified HepaRGs following a 48 h exposure at 25 μM.^98^ However, human arylacetamide deacetylase expression may be higher in femPHHs, making them more sensitive to KTC dosing in our model.

An apparent increase in 4-OH-RA (25–50 μM PFDA; *p* < 0.001) and 4-oxo-RA (25 μM PFDA; *p* < 0.01) was noted after treatment of the femPHHs with lower concentrations of PFDA (Figure 7A,B). While intriguing, the cause of this slight metabolic gain is unclear, and we are currently exploring the underlying causes of this variability. The maximum averages peaked at 25 μM PFDA for the formation of both *at*RA metabolites, deviating from the typical dose–response inhibition pattern observed in the recombinant studies for all adult retinoic acid hydroxylases tested. While the cause of this aberration may be an artifact of analytical variability within our instrument, this metabolite increase appears to be reproducible in femPHHs. One important consideration is the potential for PFDA to inhibit Phase II conjugation of 4-OH and 4-oxo-RA at lower concentrations compared to the *at*RA-metabolizing CYPs. We have limited evidence that PFAS can interfere with the activities of uridine 5′-diphospho-glucuronosyltransferases (UGTs), including those known to interact with retinoids, such as UGT1A1 (data not shown). Inhibition of UGTs at lower concentrations would likely result in a metabolic "bottleneck” limiting 4-OH and 4-oxo-RA conjugation independent of *at*RA oxidative metabolism, causing the increase compared to AC. At concentrations above the inhibition threshold for CYP26A1-mediated *at*RA oxidation, 4-OH and 4-oxo-RA are no longer produced, hence the drop in signal. Further research is necessary to verify these conjectures, ideally testing PFDA inhibition of UGT glucuronidation utilizing 4-OH and 4-oxo-RA as substrate probes for this reaction.

Despite these irregularities, there were significant dose-dependent increases in overall femPHH *at*RA levels at 50–100 μM PFDA compared to the AC (*p* < 0.001), reaching up to 182% of AC at noncytotoxic concentrations (75 μM PFDA) (Figure 7C). Moreover, the onset of *at*RA bioaccumulation in femPHHs coincides with the approximate IC_50_ values obtained for PFDA inhibition of recombinant CYP26A1 retinoic acid hydroxylase activity, reflecting a lack of hepatocellular clearance of the morphogen (Figures 3 and 7C). Metabolite formation in femPHHs was consistent with the recombinant CYP26A1 IC_50_ data at higher PFDA concentrations, in which 88.0% ± 2.2 and 83.6% ± 0.4 inhibition was recorded for 4-OH and 4-oxo-RA, respectively (80 μM PFDA) (Figure 3). Furthermore, the inhibition of *at*RA oxidation in femPHHs at higher concentrations of PFDA was compatible with the results gathered from the CYP2C8 and CYP3A4 recombinant studies in which metabolite signal was virtually absent at or above 100 and 60 μM PFDA, respectively (Figure 8). Based on the values obtained from the recombinant assays, coupled with the marked inhibition of *at*RA clearance (50–75 μM PFDA) and metabolite formation (75 μM PFDA) in the femPHHs at noncytotoxic concentrations, we can postulate that maternal hepatic *at*RA homeostasis may be vulnerable to PFDA inhibitory effects on the retinoic acid hydroxylases.

While it is established that CYP26A1 is the most prominent hepatic retinoic acid hydroxylase, the relative contributions of CYP2C8 and CYP3A4 *at*RA metabolite formation have yet to be determined in femPHHs.^27,28^ Following semi-static *at*RA exposures in adult femPHHs, CYP26A1 transcription was significantly induced in the AC compared to the VC (log_2_(FC) = 12.1; *p*_adj_ ≤ 0.05) (Supporting Information, Figure S3). Normalized relative mRNA expression of each retinoic acid hydroxylase was ranked (highest to lowest based on Fragments Per Kilobase of transcript per Million mapped reads (FPKM)) as follows: CYP3A4 ≈ CYP2C8 > CYP26A1 > CYP3A5 > CYP26B1 > CYP3A7 > CYP26C1 (data not shown). As previously reported in the primary literature, the estimated intrinsic clearance (CL_int_) of each retinoic acid hydroxylase enzyme for *at*RA, ranked highest to lowest, is as follows: CYP26A1 > CYP26C1 > CYP26B1 > CYP3A5 > CYP2C8 > CYP3A4 ≈ CYP3A7.^27,28,99^ Despite the relative mRNA expression levels in the femPHHs, CYP26A1 CL_int_ of *at*RA has been reported to reach approximately 1200 μL/min/pmol protein, a value that is estimated to sit between 10- and 10,000-fold higher than any other retinoic acid hydroxylase measured.^28,31,99^ Moreover, *at*RA binding affinities (*K*_m_) for members of the CYP26 family fall in the nanomolar range, as opposed to values in the micromolar range observed for CYP3A and CYP2C enzyme families.^30,31,48^ Given its induction following incubations with *at*RA, along with the CL_int_ and *K*_m_ values reported in the literature, we are confident that CYP26A1 is the dominant contributor to *at*RA metabolism observed in our femPHH model.

Even though *at*RA exhibits a very high affinity for CYP26A1 (*K*_m_ = 50.1 nM for 4-OH-RA formation), any dysregulation of retinoid metabolism or signaling during pregnancy can lead to irreversible consequences for the developing fetus.^13–15,31^ PFDA demonstrated the capacity to significantly perturb mRNA transcription at noncytotoxic concentrations below (50 μM) and above (75 μM) the *at*RA inhibition threshold observed in the femPHHs (Figure 9). Among the KEGG pathways affected, genes assigned to the retinol metabolism pathway (map00830) were the fifth-most downregulated in both the 50 and 75 μM groups against the AC (Figure 9A,B). The perturbation of genes involved in retinol metabolism and signaling, coupled with the inhibition of retinoic acid metabolism and clearance in the maternal liver, may lead to disrupted *at*RA gradients during critical developmental stages of embryogenesis.

The significant downregulation of *CYP26A1* (50 and 75 μM PFDA vs AC) at first appears counterintuitive, as inhibition of *at*RA metabolism may be expected to trigger an autoregulatory feedback loop in the model, in which excess retinoid induces transcription of *CYP26A1* via agonism of the RAR/RXR heterodimer (Figure 9C).^20,21^ Strictly speaking, the active-site electrostatic binding surface of CYP26A1 shares similarities with that of the RAR/RXR binding interface, as they both interact with *at*RA. This, in conjunction with the fact that the natural ligands of the nuclear hormone receptors RAR and RXR include retinoids and polyunsaturated fatty acids, gives rise to the possibility that PFDA may directly antagonize RAR/RXR receptor activity. Our RNA-sequencing data indicated that PFDA significantly downregulated the expression of *RXRα* (*RXRA*, 50 and 75 μM PFDA vs AC) and *RARβ* (*RARB*, 75 μM PFDA vs AC) (Figure 9C). There is existing evidence in mice that the craniofacial defects distinctive of retinoic acid embryopathy are facilitated via the RARβ/RXR heterodimer, which determines fusion and hypoplasia in the pharyngeal endoderm.^100^ Although PFDA has been reported as an inconclusive antagonist of RAR through luminescence-based assays, further research is necessary to delineate isoform-specific effects of the compound on RARβ signaling.^101^

It has been established that the PPAR signaling pathway itself plays a role in craniofacial development, particularly via PPARγ. A master regulator of adipogenesis, PPARγ has been shown to suppress osteoblast differentiation in mice.^102^ Upon controlling for the effects of *at*RA, PFDA significantly upregulated the transcription of *PPARγ* (*PPARG*, 50 and 75 μM PFDA vs AC) in femPHHs (Figure 9C). PFAS induction and activation of PPAR (particularly α and γ) have been extensively documented in the literature, with many reported as being dual PPARα/γ agonists, including PFOA and PFOS.^103,104^ While studies have indicated PFDA is a potent PPARα agonist, it has not been observed to interact with PPARγ, *in vitro.* ^101,105^ Transcription of PPARγ can be induced in-part by active retinoids in cell lines.^106^ However, treatment with *at*RA in the femPHH model had limited effects on *PPARG* expression (|log_2_(FC) < 1|; *p*_adj_ ≤ 0.05) (Supporting Information, Figure S3). Yet, PFDA may be capable of mimicking positive regulators of its transcription, including retinoids. We postulate that this report represents the first to identify PFDA as a potential novel inducer of PPARγ expression in primary hepatocytes. As predicted for the RAR/RXR induction pathways, our data potentiate PFDA as a receptor ligand for a variety of biological pathways. However, it is likely that the primary mechanism for the significant craniofacial abnormalities associated with developmental PFDA exposure is the result of its effects on retinol metabolism and signaling rather than its induction of PPARγ gene expression. In further support of these claims, the PPAR signaling pathway (map03320) was not among the top 10 observed up- or downregulated pathways in our KEGG enrichment analysis (Figure 9A,B).

Given the significant craniofacial abnormalities associated with prenatal PFDA exposure, the dysregulation of *WNT5B* and *WNT9A* expression in the femPHHs provides potential downstream *at*RA signaling targets to be interrogated through future experiments (Figure 9C). According to Sisson and colleagues, *wnt5b* mutant zebrafish demonstrated unique defects in craniofacial chondrocyte stacking and cartilage morphogenesis.^56^ *Wnt9a* mouse mutants have displayed various skeletal defects affecting the supra- and basioccipital bones, and the parietal bone.^57,58^ Other genes of interest involved in craniofacial development were also examined for potential dysregulation in our model, including sonic hedgehog (*SHH*) and twist family basic helix–loop–(bHLH) transcription factor 1 (*TWIST1*). However, none were significantly perturbed in the studies presented here (data not shown).

Our findings offer a potential mechanistic link between prenatal PFDA exposure and the associated craniofacial abnormalities via the dysregulation of maternal hepatic retinoic acid metabolism and signaling during fetal development. Further research will be conducted *in vivo* to validate the relationship between PFDA and *at*RA homeostasis and evaluate PFDA as an inhibitor of CYP26A1 in chordates. In the Shanghai Maternal-Child Pairs Cohort, consisting of 1076 participants, the detection rate of PFDA was >90% in maternal serum and >50% in cord serum across all trimesters.^107^ PFDA is widely implemented in stain- and grease-proof coatings, textiles, furniture, and carpet.^108^ Here, we observed clear evidence of the propensity of PFDA to inhibit the catalytic activity of retinoic acid hydroxylases in the maternal liver, potentially hindering the first line of fetal defense against the disruption of *at*RA homeostasis during key developmental milestones. The CYP26A1 homology model displayed significant overlap between the *at*RA and PFDA binding sites in their interactions with the heme iron and predicted residue contacts. We also demonstrated the propensity for PFDA to dysregulate the expression of genes involved in retinol metabolism and signaling in femPHHs. We posit that PFDA has the potential to serve as a low-affinity ligand for a variety of targets impacting human neonatal development. Therefore, PFDA bears the unique structural disposition to mimic and antagonize retinoids at critical morphogenic windows in vulnerable populations, offering a novel mechanism of PFAS teratogenicity to be further explored.

## Supplementary Material



## Notes

Recombinant CYP26B1 *at*RA oxidative activity is resilient to PFAS (Figure S1), images of femPHHs following retinoid and PFDA exposures (Figure S2), enriched KEGG pathways and analysis of differentially expressed genes in femPHHs exposed to *at*RA (Figure S3), index of differentially expressed genes utilized in analysis (Table S1) . (PDF)

## Abbreviations

PFAS: per- and polyfluoroalkyl substances
CYP: cytochrome P450
DHEA-S: dehydroepiandrosterone sulfate
*at*RA: all-*trans*-retinoic acid
9-*cis*-RA: 9-*cis*-retinoic acid
13-*cis*-RA: 13-*cis*-retinoic acid
RAR: retinoic acid receptor
RXR: retinoid X receptor
RAREs: retinoic acid response elements
4-OH-RA: 4-hydroxy-retinoic acid
4-oxo-RA: 4-oxo-retinoic acid
IS: internal standard
4-oxo-RA-*d*_3_: 4-oxo-retinoic acid-(9-methyl)-*d*_3_
POPs: persistent organic pollutants
EDCs: endocrine-disrupting chemicals
PFOA: perfluorooctanoic acid
PFOS: perfluorooctanesulfonic acid
PFNA: perfluorononanoic acid
PFDA: perfluorodecanoic acid
PFDS: perfluorodecanesulfonic acid
PFUnDA: perfluoroundecanoic acid
FBSA: perfluorobutane sulfonamide
PFBS: perfluorobutanesulfonic acid
PFPeA: perfluoropentanoic acid
GenX: ammonium perfluoro­(2-methyl-3-oxahexanoate
FHxSA: perfluorohexanesulfonamide
PFHxS: perfluorohexanesulfonic acid
4:2 FTS: 1*H*,1*H*,2*H*,2*H*-perfluorohexanesulfonic acid
LC-MS/MS: liquid chromatography-tandem mass spectrometry
IC50: half-maximal inhibitory concentration
femPHHs: female primary human hepatocytes
TAL: talarozole
KTC: ketoconazole
BHT: 2,6-di-*tert*-butyl-4-methylphenol
NADP+: β-nicotinamide adenine dinucleotide phosphate
Pen/Strep: penicillin/streptomycin
DMSO: dimethyl sulfoxide
ACN: acetonitrile
HPLC: high-performance liquid chromatography
LDH: lactate dehydrogenase (LDH)
BSA: bovine serum albumin
PBS: phosphate-buffered saline
AC: atRA control
VC: vehicle control
RNA: ribonucleic acid
mRNA: mRNA
DEGs: differentially expressed genes
KEGG: Kyoto Encyclopedia of Genes and Genomes
UPLC: ultra-performance liquid chromatography
ESI: electrospray ionization
MEM: multiple reaction monitoring
CE: collision energy
CV: cone voltage
BH: Benjamini–Hochberg
FDR: false discovery rate
FC: fold change
SD: standard deviation
ns: not significant
PFLs: palpebral fissure lengths
BMD10: benchmark dose corresponding to 10% increased risk
DNBC: Danish National Birth Cohort
VAD: vitamin A deficiency
CLint: intrinsic clearance
FPKM: Fragments Per Kilobase of transcript per Million mapped reads
SHH: sonic hedgehog
bHLH: basic helix–loop–helix
TWIST1: twist family basic helix–loop–helix transcription factor 1
