# Supplementary material for: New Mechanistic Evidence for Perfluorodecanoic Acid (PFDA) Teratogenicity via CYP26A1-Mediated Retinoic Acid Metabolism and Signaling
Source: Chem Res Toxicol. 2026 Mar 30;39(4):566–83. doi: 10.1021/acs.chemrestox.5c00468 (PMC13100982; doi:10.1021/acs.chemrestox.5c00468)
Supplement: Supplementary file 1 [file tx5c00468_si_001.pdf]

SUPPORTING INFORMATION

New mechanistic evidence for perfluorodecanoic acid (PFDA) teratogenicity via CYP26A1-mediated retinoic acid metabolism and signaling

*Michaela Hvizdak, Sylvie E. Kandel, and Jed N. Lampe**

Department of Pharmaceutical Sciences, Skaggs School of Pharmacy, University of Colorado, Aurora, Colorado 80045, United States.

* Corresponding author; please direct all correspondence to: jed.lampe@cuanschutz.edu

Table of Contents **Page**

Figure S1. Recombinant CYP26B1 *at*RA oxidative activity is resilient to PFAS. ……………….S3

Figure S2. Images of femPHHs following retinoid and PFDA exposures. ……………………....S4

Figure S3. Enriched KEGG pathways and analysis of differentially expressed genes in femPHHs exposed to *at*RA. ……………………………………………………………..………..…S5

Table S1. Index of differentially expressed genes utilized in analysis. ………………………..…S7

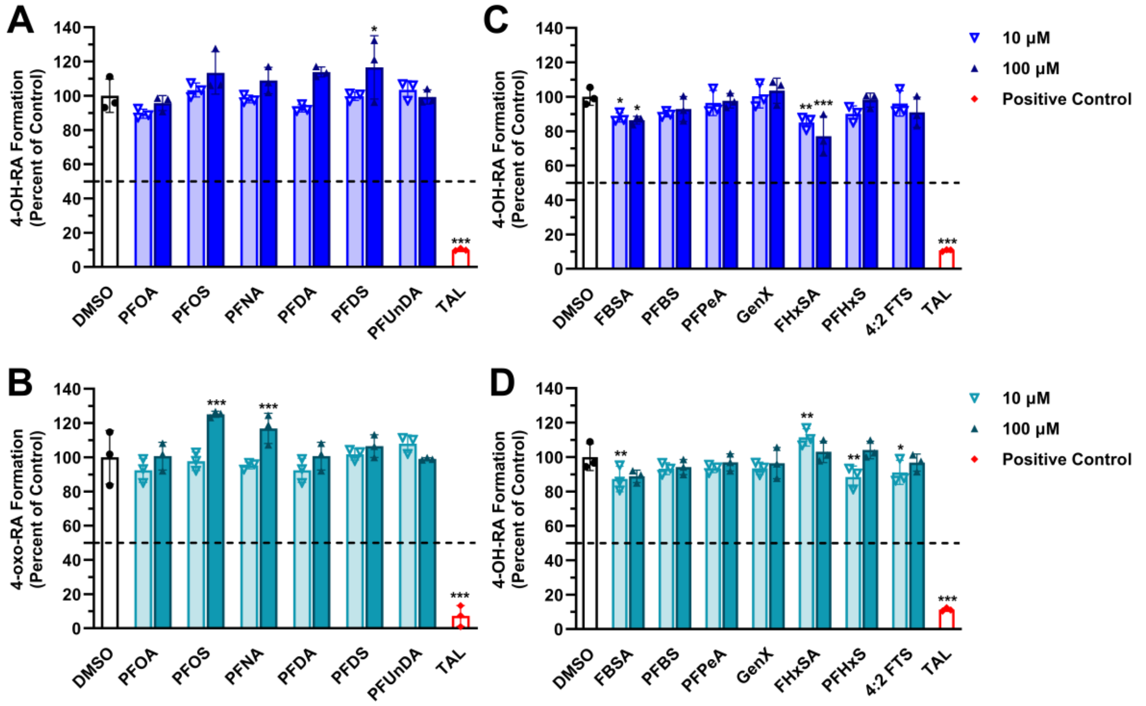

Figure S1. Recombinant CYP26B1 *at*RA oxidative activity is resilient to PFAS. Triplicate values of CYP26B1 4-OH (A and C), and 4-oxo-RA (B and D) percent of control following incubation with long-chain (PFOA, PFOS, PFNA, PFDA, PFDS, and PFUnDA; A and B), and short-chain (FBSA, PFBS, PFPeA, GenX, FHxSA, PFHxS, and 4:2 FTS; C and D) PFAS. Talarozole (TAL; 2 µM) was utilized as the positive control for inhibition. Data represented as mean ± SD. Statistical significance against the DMSO control indicated by * $p < 0.05$, ** $p < 0.01$, *** $p < 0.001$; two-way ANOVA and Dunnett's post-hoc test.

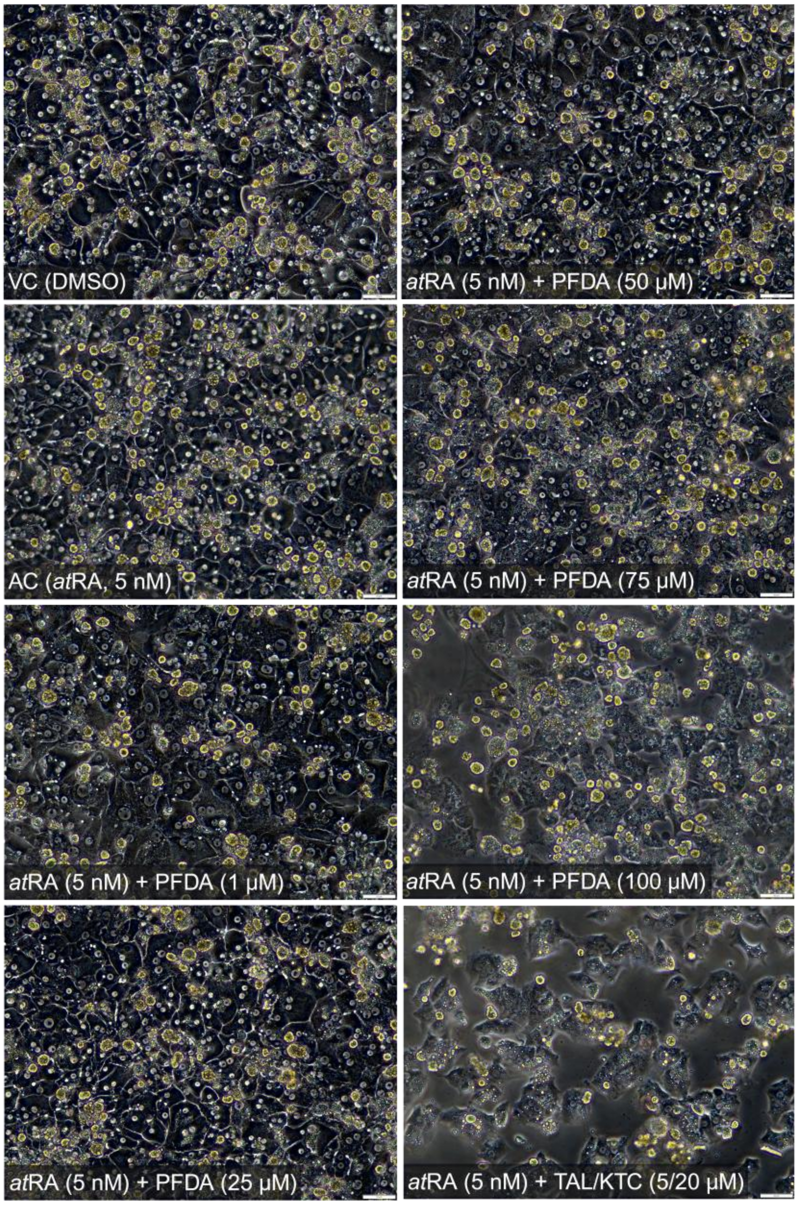

Figure S2. Images of femPHHs following retinoid and PFDA exposures. femPHHs were dosed daily with the vehicle control (VC; DMSO), *at*RA control (AC; 5 nM), or atRA (5 nM) plus PFDA (1-100 μM) or the inhibitor cocktail (talarozole/ketoconazole, TAL/KTC; 5 and 20 μM, respectively). Images depict morphological changes observed in the femPHHs dosed with *at*RA (5 nM) plus PFDA (100 μM) or TAL/KTC after 48-h. Images taken at 20x magnification. Scale bar indicates 50 μm resolution.

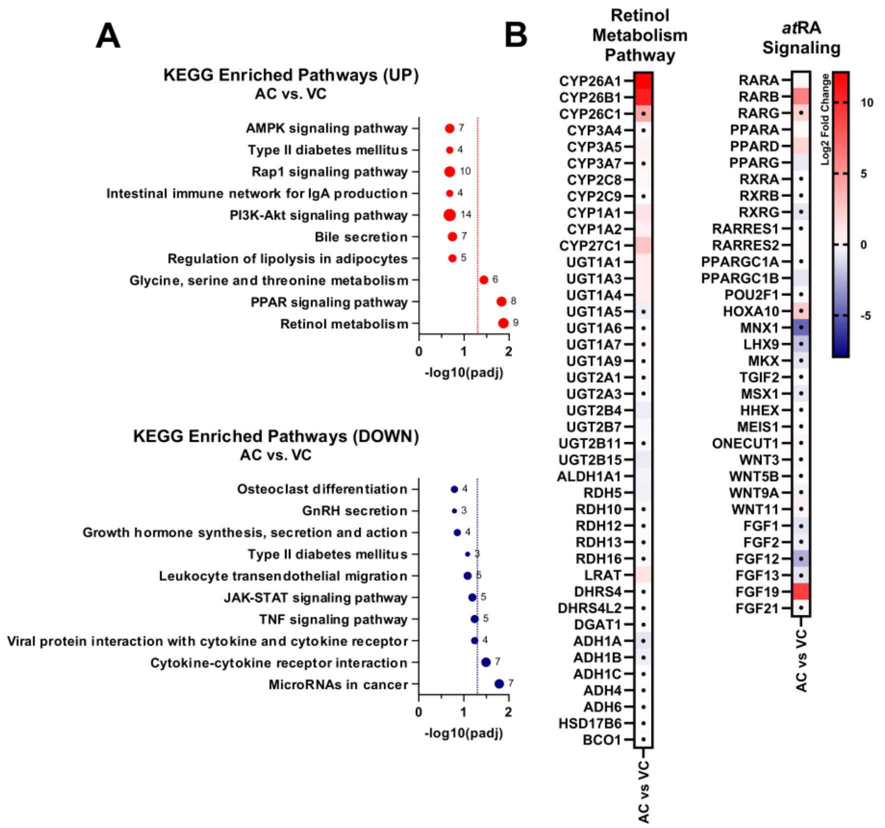

Figure S3. Enriched KEGG pathways and analysis of differentially expressed genes in femPHHs exposed to *at*RA. Female primary human hepatocytes (femPHHs) were dosed with *at*RA (5 nM) for 48 h, followed by a 4 h *at*RA spike (3 µM). (A) Enriched up- (red) and down-regulated (blue) KEGG pathways in the *at*RA control (AC) ranked based on significance scaled as $-\log_{10}(p_{adj})$ on the horizontal axis. Point size and the adjacent labels correspond to the number of genes annotated to a specific KEGG pathway. Statistical significance ($p_{adj} < 0.05$) against the vehicle control (VC) indicated by each dashed line. (B) Heatmaps of differentially expressed genes within specific KEGG pathways, including those related to the retinol metabolism pathway, and downstream targets of atRA signaling. The color scale represents the $\log_2$(fold change) expression. The threshold of significant differential expression is "$|\log_2(\text{fold change})| \geq 1.0$ and adjusted p-value (p_{adj}) ≤ 0.05". Adjusted p-values were determined using the Benjamini-

Hochberg (BH) correction for false discovery rate (FDR). Log_2(fold change) values with p_{adj} > 0.05 denoted with a dot "●". Log_2(fold change) values with p_{adj} > 0.05 denoted with a dot "●". Statistical analysis was performed by Novogene Corporation, Inc. (Sacramento, CA).

Table S1. Index of differentially expressed genes utilized in analysis. Alphabetized list of DEGs utilized in RNA-sequencing analysis for femPHHs. Gene names are accompanied by descriptions and respective Ensembl ID numbers.

Gene Name	Description	Ensembl ID
ALDH1A1	aldehyde dehydrogenase 1 family member A1 [Source:HGNC Symbol;Acc:HGNC:402]	ENSG00000165092
CYP1A1	cytochrome P450 family 1 subfamily A member 1 [Source:HGNC Symbol;Acc:HGNC:2595]	ENSG00000140465
CYP1A2	cytochrome P450 family 1 subfamily A member 2 [Source:HGNC Symbol;Acc:HGNC:2596]	ENSG00000140505
CYP26A1	cytochrome P450 family 26 subfamily A member 1 [Source:HGNC Symbol;Acc:HGNC:2603]	ENSG00000095596
CYP26B1	cytochrome P450 family 26 subfamily B member 1 [Source:HGNC Symbol;Acc:HGNC:20581]	ENSG00000003137
CYP26C1	cytochrome P450 family 26 subfamily C member 1 [Source:HGNC Symbol;Acc:HGNC:20577]	ENSG00000187553
CYP27C1	cytochrome P450 family 27 subfamily C member 1 [Source:HGNC Symbol;Acc:HGNC:33480]	ENSG00000186684
CYP2C8	cytochrome P450 family 2 subfamily C member 8 [Source:HGNC Symbol;Acc:HGNC:2622]	ENSG00000138115
CYP2C9	cytochrome P450 family 2 subfamily C member 9 [Source:HGNC Symbol;Acc:HGNC:2623]	ENSG00000138109
CYP3A4	cytochrome P450 family 3 subfamily A member 4 [Source:HGNC Symbol;Acc:HGNC:2637]	ENSG00000160868
CYP3A5	cytochrome P450 family 3 subfamily A member 5 [Source:HGNC Symbol;Acc:HGNC:2638]	ENSG00000106258
CYP3A7	cytochrome P450 family 3 subfamily A member 7 [Source:HGNC Symbol;Acc:HGNC:2640]	ENSG00000160870

DGAT1	diacylglycerol O-acyltransferase 1 [Source:HGNC Symbol;Acc:HGNC:2843]	ENSG00000185000
DHRS4	dehydrogenase/reductase 4 [Source:HGNC Symbol;Acc:HGNC:16985]	ENSG00000157326
DHRS4L2	dehydrogenase/reductase 4 like 2 [Source:HGNC Symbol;Acc:HGNC:19731]	ENSG00000187630
FGF1	fibroblast growth factor 1 [Source:HGNC Symbol;Acc:HGNC:3665]	ENSG00000113578
FGF12	fibroblast growth factor 12 [Source:HGNC Symbol;Acc:HGNC:3668]	ENSG00000114279
FGF13	fibroblast growth factor 13 [Source:HGNC Symbol;Acc:HGNC:3670]	ENSG00000129682
FGF19	fibroblast growth factor 19 [Source:HGNC Symbol;Acc:HGNC:3675]	ENSG00000162344
FGF2	fibroblast growth factor 2 [Source:HGNC Symbol;Acc:HGNC:3676]	ENSG00000138685
FGF21	fibroblast growth factor 21 [Source:HGNC Symbol;Acc:HGNC:3678]	ENSG00000105550
HHEX	hematopoietically expressed homeobox [Source:HGNC Symbol;Acc:HGNC:4901]	ENSG00000152804
HOXA10	homeobox A10 [Source:HGNC Symbol;Acc:HGNC:5100]	ENSG00000253293
LHX9	LIM homeobox 9 [Source:HGNC Symbol;Acc:HGNC:14222]	ENSG00000143355
LRAT	lecithin retinol acyltransferase [Source:HGNC Symbol;Acc:HGNC:6685]	ENSG00000121207
MEIS1	Meis homeobox 1 [Source:HGNC Symbol;Acc:HGNC:7000]	ENSG00000143995
MKX	mohawk homeobox [Source:HGNC Symbol;Acc:HGNC:23729]	ENSG00000150051

MNX1	motor neuron and pancreas homeobox 1 [Source:HGNC Symbol;Acc:HGNC:4979]	ENSG00000130675
MSX1	msh homeobox 1 [Source:HGNC Symbol;Acc:HGNC:7391]	ENSG00000163132
ONECUT1	one cut homeobox 1 [Source:HGNC Symbol;Acc:HGNC:8138]	ENSG00000169856
POU2F1	POU class 2 homeobox 1 [Source:HGNC Symbol;Acc:HGNC:9212]	ENSG00000143190
PPARA	peroxisome proliferator activated receptor alpha [Source:HGNC Symbol;Acc:HGNC:9232]	ENSG00000186951
PPARD	peroxisome proliferator activated receptor delta [Source:HGNC Symbol;Acc:HGNC:9235]	ENSG00000112033
PPARG	peroxisome proliferator activated receptor gamma [Source:HGNC Symbol;Acc:HGNC:9236]	ENSG00000132170
PPARGC1A	PPARG coactivator 1 alpha [Source:HGNC Symbol;Acc:HGNC:9237]	ENSG00000109819
PPARGC1B	PPARG coactivator 1 beta [Source:HGNC Symbol;Acc:HGNC:30022]	ENSG00000155846
RARA	retinoic acid receptor alpha [Source:HGNC Symbol;Acc:HGNC:9864]	ENSG00000131759
RARB	retinoic acid receptor beta [Source:HGNC Symbol;Acc:HGNC:9865]	ENSG00000077092
RARG	retinoic acid receptor gamma [Source:HGNC Symbol;Acc:HGNC:9866]	ENSG00000172819
RARRES1	retinoic acid receptor responder 1 [Source:HGNC Symbol;Acc:HGNC:9867]	ENSG00000118849
RARRES2	retinoic acid receptor responder 2 [Source:HGNC Symbol;Acc:HGNC:9868]	ENSG00000106538
RDH10	retinol dehydrogenase 10 [Source:HGNC Symbol;Acc:HGNC:19975]	ENSG00000121039

RDH12	retinol dehydrogenase 12 [Source:HGNC Symbol;Acc:HGNC:19977]	ENSG00000139988
RDH13	retinol dehydrogenase 13 [Source:HGNC Symbol;Acc:HGNC:19978]	ENSG00000160439
RDH16	retinol dehydrogenase 16 [Source:HGNC Symbol;Acc:HGNC:29674]	ENSG00000139547
RDH5	retinol dehydrogenase 5 [Source:HGNC Symbol;Acc:HGNC:9940]	ENSG00000135437
RXRA	retinoid X receptor alpha [Source:HGNC Symbol;Acc:HGNC:10477]	ENSG00000186350
RXRB	retinoid X receptor beta [Source:HGNC Symbol;Acc:HGNC:10478]	ENSG00000204231
RXRG	retinoid X receptor gamma [Source:HGNC Symbol;Acc:HGNC:10479]	ENSG00000143171
TGIF2	TGFB induced factor homeobox 2 [Source:HGNC Symbol;Acc:HGNC:15764]	ENSG00000118707
UGT1A1	UDP glucuronosyltransferase family 1 member A1 [Source:HGNC Symbol;Acc:HGNC:12530]	ENSG00000241635
UGT1A3	UDP glucuronosyltransferase family 1 member A3 [Source:HGNC Symbol;Acc:HGNC:12535]	ENSG00000243135
UGT1A4	UDP glucuronosyltransferase family 1 member A4 [Source:HGNC Symbol;Acc:HGNC:12536]	ENSG00000244474
UGT1A5	UDP glucuronosyltransferase family 1 member A5 [Source:HGNC Symbol;Acc:HGNC:12537]	ENSG00000240224
UGT1A6	UDP glucuronosyltransferase family 1 member A6 [Source:HGNC Symbol;Acc:HGNC:12538]	ENSG00000167165
UGT1A7	UDP glucuronosyltransferase family 1 member A7 [Source:HGNC Symbol;Acc:HGNC:12539]	ENSG00000244122
UGT1A9	UDP glucuronosyltransferase family 1 member A9 [Source:HGNC Symbol;Acc:HGNC:12541]	ENSG00000241119

UGT2A1	UDP glucuronosyltransferase family 2 member A1 complex locus [Source:HGNC Symbol;Acc:HGNC:12542]	ENSG00000173610
UGT2A3	UDP glucuronosyltransferase family 2 member A3 [Source:HGNC Symbol;Acc:HGNC:28528]	ENSG00000135220
UGT2B11	UDP glucuronosyltransferase family 2 member B11 [Source:HGNC Symbol;Acc:HGNC:12545]	ENSG00000213759
UGT2B15	UDP glucuronosyltransferase family 2 member B15 [Source:HGNC Symbol;Acc:HGNC:12546]	ENSG00000196620
UGT2B4	UDP glucuronosyltransferase family 2 member B4 [Source:HGNC Symbol;Acc:HGNC:12553]	ENSG00000156096
UGT2B7	UDP glucuronosyltransferase family 2 member B7 [Source:HGNC Symbol;Acc:HGNC:12554]	ENSG00000171234
WNT11	Wnt family member 11 [Source:HGNC Symbol;Acc:HGNC:12776]	ENSG00000085741
WNT3	Wnt family member 3 [Source:HGNC Symbol;Acc:HGNC:12782]	ENSG00000108379
WNT5B	Wnt family member 5B [Source:HGNC Symbol;Acc:HGNC:16265]	ENSG00000111186
WNT9A	Wnt family member 9A [Source:HGNC Symbol;Acc:HGNC:12778]	ENSG00000143816
